# Pharmacological inhibition of MYC to mitigate chemoresistance in preclinical models of squamous cell carcinoma

**DOI:** 10.7150/thno.88759

**Published:** 2024-01-01

**Authors:** Shuo Liu, Zhen Qin, Yaqing Mao, Ning Wang, Wenbo Zhang, Yujia Wang, Yiwen Chen, Lingfei Jia, Xin Peng

**Affiliations:** Department of Oral and Maxillofacial Surgery, Peking University School and Hospital of Stomatology, Beijing 100081, China. National Center of Stomatology & National Clinical Research Center for Oral Diseases & National Engineering Laboratory for Digital and Material Technology of Stomatology, Beijing 100081, China.

**Keywords:** Cisplatin resistance, Cancer stem cells, MYC, Head and neck squamous cell carcinoma, DNA damage response

## Abstract

**Rationale:** Cisplatin-based chemotherapy is the first-line treatment for late-stage head and neck squamous cell carcinoma (HNSCC). However, resistance to cisplatin has become a major obstacle for effective therapy. Cancer stem cells (CSCs) are critical for tumor initiation, growth, metastasis, and chemoresistance. How to effectively eliminate CSCs and overcome chemoresistance remains a key challenge. Herein, we confirmed that MYC plays critical roles in chemoresistance, and explored targeting MYC to overcome cisplatin resistance in preclinical models.

**Methods:** The roles of MYC in HNSCC cisplatin resistance and cancer stemness were tested *in vitro* and *in vivo*. The combined therapeutic efficiency of MYC targeting using the small molecule MYC inhibitor MYCi975 and cisplatin was assessed in a 4‑nitroquinoline 1-oxide-induced model and in a patient-derived xenograft model.

**Results:** MYC was highly-expressed in cisplatin-resistant HNSCC. Targeting MYC using MYCi975 eliminated CSCs, prevented metastasis, and overcame cisplatin resistance. MYCi975 also induced tumor cell-intrinsic immune responses, and promoted CD8^+^ T cell infiltration. Mechanistically, MYCi975 induced the DNA damage response and activated the cGAS-STING-IRF3 signaling pathway to increase CD8^+^ T cell-recruiting chemokines.

**Conclusions:** Our findings suggested that targeting MYC might eliminate CSCs, prevent metastasis, and activate antitumor immunity to overcome cisplatin resistance in HNSCC.

## Introduction

Head and neck squamous cell carcinoma (HNSCC), developing from the oral cavity, oropharynx, larynx, and hypopharynx, is a highly malignant tumor with poor prognosis, which frequently metastasizes to cervical lymph nodes 1. Current treatment, including surgical resection, radiation therapy, chemotherapy, or their combinations, represent the classical options for managing HNSCC 2, 3. However, the 5-year survival rate for patients with HNSCC has not increased over past decades because of lymph node metastasis, local recurrence, and the high incidence of therapeutic resistance 4-6. Therefore, identifying the cellular and molecular mechanisms of recurrence and developing novel effective therapies is urgently required.

At present, cisplatin-based chemotherapy remains the most effective first-line agent to treat recurrent and metastatic disease 7. Although HNSCC responds initially to chemotherapy, many patients rapidly develop chemoresistance and eventually experience recurrence. Thus, overcoming cisplatin resistance remains a critical goal for anticancer therapy and considerable efforts have been undertaken to solve this problem throughout the past three decades 8. The complex mechanisms of cisplatin resistance involve cancer stem cells (CSCs), autophagy, epithelial-mesenchymal transition, drug efflux, and metabolic reprogramming 8-10. CSCs are critical for tumor initiation and growth, and might be associated with metastasis and recurrence. Growing evidence suggests that CSCs are responsible for cancer therapy resistance and relapse or recurrence 11-14. A number of studies have focused on the inhibition of regulatory pathways that are critical for the stemness and tumorigenic potential of CSCs 15-18. Recently, using *in vivo* lineage tracing, we showed that CSCs have vital functions in chemotherapy resistance, recurrence, and metastasis of HNSCC 18, 19. However, little is known about how cancer stemness is regulated molecularly and epigenetically.

In this study, we identified that MYC, which plays critical roles in tumorigenesis and therapeutic resistance 20, was highly expressed in a cisplatin-resistant patient-derived xenograft (PDX) model of HNSCC and regulates cancer stemness. Targeting MYC using a small molecule inhibitor (MYCi975) not only eliminated CSCs, prevented metastasis, and overcame cisplatin resistance, but also promoted CD8^+^ T cell intratumor infiltration in HNSCC treated with cisplatin. Our preclinical studies provide an important foundation to develop a new clinical trial for cisplatin-based combination therapy using MYC inhibitors.

## Methods

### Human HNSCC samples

The use of human HNSCC samples in this study was approved by the Ethics Committee of Peking University School and Hospital of Stomatology (PKUSSIRB-2012010). Fresh human HNSCC primary tissues were obtained from Peking University Hospital of Stomatology and subcutaneously inoculated into the flanks of 6-8 weeks old Nonobese diabetic/severe combined immunodeficiency (NOD/SCID) mice, which lack functional T cells and B cells, and show lymphopenia and hypogammaglobulinemia, but have a normal hematopoietic microenvironment.

### Mice

*Bmi1^CreER^* (JAX: 010531) and *Rosa^tdTomato^* (JAX: 007909) mouse strains were crossbred to generate *Bmi1^CreER^*; *Rosa^tdTomato^* mice. *K14^CreER^* (NM-KI-190024) and *Myc^flox/flox^* (NM-CKO-210184) mouse strains were crossbred to generate *K14^CreER^*; *Myc^flox/flox^*. *Bmi1^CreER^* and *Rosa^tdTomato^* mice were purchased from The Jackson Laboratory (Bar Harbor, ME, USA). *K14^CreER^* and *Myc^flox/flox^* were purchased from Shanghai Model Organisms Center, Inc. (Shanghai, China). The NOD-SCID mice, and BALB/c-nude female mice were purchased from Beijing Sibeifu Biotechnology Co. Ltd (Beijing, China). All the above mice were housed under specific-pathogen-free (SPF) conditions. All animal studies were performed in compliance with the regulations and the Peking University institutional animal care guidelines.

### HNSCC PDX Model

Subcutaneous inoculation of human HNSCC primary tissues into the flanks of 6 - 8-week-old NOD-SCID mice was used to generate the PDX models. The patients used to generate the PDX models did not receive cisplatin treatment. Cisplatin‑resistant PDX models were established as described previously 21. Briefly, 10 independent PDX models were given cisplatin (3 mg/kg) for 4-6 cycles. Cisplatin (Selleck, Houston, TX, USA; Cat# S1166) was dissolved in saline. We observed a spectrum of responses: Eight out of ten models showed a broad index of partial responses, ranging from 55% to 80% tumor growth inhibition, while two showed 30-40% tumor growth inhibition, without any dose-limiting toxicity, as measured by animal weights. To ensure that tumors progressing through multiple cycles of chemotherapy would develop intrinsic chemoresistance, the tumors of two cases of PDX model that showed the lowest response to cisplatin therapy were disaggregated, re-implanted into a second generation of mice, and selected again through 4-6 cycles of cisplatin therapy. Following this, we established two cisplatin‑resistant PDX models.

MYCi975 (MedMol, Shanghai, China; Cat# S89011) was dissolved in 5% dimethyl sulfoxide (DMSO) in corn oil. For the cisplatin-resistant PDX mice, two weeks after tumor implantation, the mice were divided into four groups and given: 1) control vehicle; 2) cisplatin (3 mg/kg body weight once a week); 3) MYCi975 (100 mg/kg, every 2 days); and 4) MYCi975 plus cisplatin for 6 weeks. Thereafter, the mice were sacrificed and the tumor samples were dissected and isolated immediately. The tumor volume was determined using the volume formula for an ellipsoid: 1/2 × D × d^2^, where D is the longer diameter and d is the shorter diameter.

### Subcutaneous dorsal cell line-derived xenograft model

Cisplatin-resistant HN6-R cells (5 × 10^6^) transfected by shMYC or shCtrl and Matrigel were injected into the subcutaneous dorsal region of BALB/c-nude mice. One week after injection, the mice were divided into difference groups, and given vehicle or cisplatin (3 mg/kg body weight once a week) for 4 weeks. Thereafter, the mice were sacrificed and the tumor samples were dissected and isolated immediately. The tumor volume was determined using the volume formula for an ellipsoid: 1/2 × D × d^2^, where D is the longer diameter and d is the shorter diameter.

### 4-nitroquinoline 1-oxide mouse model of HNSCC, treatment and histology

For the induction of HNSCC, six-week-old mice were treated with drinking water containing 50 µg/mL 4-nitroquinoline 1-oxide (4NQO) (Santa Cruz Biotechnology, Santa Cruz, CA, USA; Cat# 256815) for 16 weeks for tumor formation and lymph node metastasis and then given normal drinking water. The mice were divided randomly into different groups at 22 weeks. For lineage tracing and *Myc* knockout studies, mice were intraperitoneally injected with tamoxifen (9 mg per 40 g body weight; Sigma-Aldrich, St. Louis, MO, USA; Cat# T5648).

For treatment, the tumor-bearing mice were divided randomly into four groups and given: 1) control vehicle; 2) cisplatin (3 mg/kg body weight once a week); 3) MYCi975 (100 mg/kg, every 2 days); and 4) MYCi975 plus cisplatin for 4 weeks. After the mice were sacrificed, their cervical lymph nodes and tongues were harvested immediately, and the lesion surface areas were measured. For histological analysis and immunostaining, longitudinally cut tongues (dorsal/ventral) and intact lymph nodes were fixed overnight in 10% buffered formalin and embedded in paraffin. Tissue blocks were cut into 10-16 sections at 4 μm thickness and stained with hematoxylin and eosin (H&E). The SCC number was counted and areas were measured as described previously 15. HNSCC invasiveness was scored based on the following criteria: showing signs of normal or epithelial dysplasia appearance (grade 1); distinct invasion, unclear basement membrane, and diffuse infiltration into the superficial portion of the muscle layer (grade 2); and loss of the basement membrane, extensive invasion into the deep muscle layer (grade 3). To assess lymph node metastasis, the sections of cervical lymph nodes were immunostained using anti‑pan-cytokeratin (PCK) antibodies (Santa Cruz Biotechnology, Cat# sc-8018, 1:200), which specifically detected epithelial tumor cells in lymph nodes.

To test MYCi975 and cisplatin tolerability *in vivo*, the major organs were collected for histopathological analysis and blood was also collected for routine blood and biochemical index analysis.

### Cell culture, MYC knockdown, and drug treatment

Human HNSCC cell lines, HN6 and SCC15, were obtained from the American Type Culture Collection (ATCC, Manassas, VA, USA). These cells were grown in Dulbecco's modified Eagle's medium (DMEM) containing 10% fetal bovine serum. Cisplatin-resistant HN6-R and SCC15-R cell lines were established using a gradient increase in the cisplatin dose and were maintained in 3 μM cisplatin, as described previously 15. The IC50 and EC50 of the parental and resistant cells were calculated. All the cells were tested and were not contaminated with mycoplasmas.

The lentiviruses used for *MYC* knockdown (KD) were purchased from Integrated Biotech Solutions Co., Ltd (Shanghai, China). Briefly, the scramble control or the specific *MYC* KD (shMYC, a short hairpin RNA)) lentiviral plasmid were cotransfected into HEK293T cells together with two helper plasmids psPAX2 (Addgene, Watertown, MA, USA Cat# 12260) and pMD2.G (Addgene, Cat# 12259). Viral supernatants were harvested for cell infection at 72 h after transfection. Cells were infected with lentiviruses in the presence of polybrene (Sigma-Aldrich, Shanghai, China, Cat# H9268), selected with puromycin (Sigma-Aldrich, Cat# P9620), and expanded before being used for subsequent assays.

For drug administration, cisplatin was dissolved in saline and MYCi975 was dissolved in DMSO, and were added to the culture medium when the cells reached 70-80% confluence.

### TUNEL experiment

To detect the apoptosis in HNSCC cells, TUNEL (terminal deoxynucleotidyl transferase dUTP nick end labeling) positivity was assessed using a TUNEL apoptosis detection kit (Solarbio, Beijing, China; Cat# T2196) according to the manufacturer's instructions. Images were then captured under a fluorescence microscope (Olympus, Tokyo, Japan).

### Cell proliferation assay

Cell proliferation was measured *in vitro* using the Cell Counting Kit-8 (CCK-8) assay (Beyotime; Jiangsu, China; Cat# C0037). Briefly, 3 × 10^3^ cells per well were added into 96-well plates with DMEM containing 10% FBS. Every 24 h, 100 μl of CCK-8 solution (1:10 dilution) was added to each well, and the cultures were incubated for 1 h at 37 °C. Color development was quantified photometrically at 450 nm using an ELx808 absorbance microplate reader (BioTeK Instruments, Winooski, VT, USA).

### CSCs isolation and flow cytometry

To isolate CSCs from HNSCC PDXs, mice were euthanized and tumors were dissected and isolated. Tumors were chopped into small pieces using scalpels in petri dishes on ice, and then dissociated into a single cell suspension using a Tumor Dissociation Kit (Miltenyi Biotec, Bergisch Gladbach, Germany; Cat# 130-095-929). Then, the single cell suspension was stained using an ALDHEFLUOR assay kit (STEMCELL Technologies, Vancouver, Canada; Cat# 01700) to label the alcohol dehydrogenase (ALDH)^high^ populations. The single cell suspension was incubated with anti-epithelial cell adhesion molecule (EpCAM)-phycoerythrin (PE) antibodies (Miltenyi Biotec, Cat# 130-111-116, 1:100) for 30 min at room temperature and then sorted by fluorescence activated cell sorting (FACS). The results were analyzed using FlowJo software (Treestar Inc., Ashland, OR, USA).

### Tumorsphere formation assay

For the tumorsphere formation assay, FACS-sorted cells were seeded in ultra-low attachment plates and cultured in serum-free DMEM/F12 (Thermo Fisher Scientific, Waltham, MA, USA; Cat# 11330-032) supplemented with 1% B27 supplement (Thermo Fisher Scientific, Cat# 17504044), 1% N2 supplement (Thermo Fisher Scientific, Cat# 17502048), antibiotics (streptomycin and penicillin, 100 mg/mL; Thermo Fisher Scientific, Cat# 15140122), human recombinant epidermal growth factor (EGF; 20 ng/mL; R&D Systems, Minneapolis, MN, USA; Cat# 236-EG-01M), and human recombinant basic fibroblast growth factor (bFGF; 10 ng/mL; R&D Systems, Cat# 233-FB-025/CF), in an incubator at 37 °C with a 5% CO_2_ atmosphere.

### Extreme Limiting-dilution assay and the orthotopic HNSCC model

For *in vivo* extreme limiting-dilution transplantation in nude mice, HN6-derived ALDH^high^ CSC-like cells or EpCAM^+^ ALDH^high^ CSCs were mixed with Matrigel and subcutaneously injected into nude mice. Then, the mice were divided into two groups, and given vehicle or MYCi975 (100 mg/kg, every 2 days). The Extreme Limiting Dilution Analysis software was used to analyze the data, as described previously 22.

For the orthotopic nude mouse model of HNSCC, EpCAM^+^ ALDH^high^ CSCs from HNSCC PDX were injected into the tongues of nude mice. One week after injection, the mice were divided into four groups and given: 1) control vehicle; 2) cisplatin (3 mg/kg body weight once a week); 3) MYCi975 (100 mg/kg, every 2 days); and 4) MYCi975 plus cisplatin for 4 weeks. After the mice were sacrificed, their cervical lymph nodes and tongues were harvested immediately. Then, the tumor volume was computed using the formula for an ellipsoid: 1/2 × D × d^2^, where D is the longer diameter and d is the shorter diameter.

### Immunostaining

Immunohistochemical staining was performed on 4 μm-thick sections from 10% buffered formalin fixed, paraffin-embedded samples. Sections were incubated with the following primary antibodies at 4 °C overnight: anti-MYC (Cell Signaling Technology, Danvers, MA, USA; Cat# 18583, 1:100) and anti-PCK (Santa Cruz Biotechnology, Cat# sc-8018, 1:200). Then, the samples were stained with horseradish peroxidase-labelled secondary antibody (ZSGB-BIO, Beijing, China; Cat# PV-6001) for 1 h at room temperature. The signals were detected using 3,3′-Diaminobenzidine (DAB) as a chromogen (ZSGB-BIO, Cat# ZLI-9018), followed by counterstaining using hematoxylin. All images were acquired under an optical microscope (Olympus).

For immunofluorescent staining, sections were stained with the following primary antibodies: anti-CD8α (Cell Signaling Technology, Cat# 98941, 1:200), anti-PCK (Abcam, Cat# ab9377, 1:200), anti-phospho-Histone H2A.X (Cell Signaling Technology, Cat# 9718, 1:400), and anti-phosphorylated interferon regulatory factor 3 (p‑IRF3) (Cell Signaling Technology; Cat# 37829; 1:400) overnight at 4 °C. The immunocomplexes were detected and visualized using related secondary antibodies conjugated with cyanine (Cy)2 or Cy3 (Jackson ImmunoResearch Laboratories, West Grove, PA, USA). Sections were then counterstained with 4'6'‑diamidino‑2‑phenilindole (DAPI, Solarbio, Cat# C0060) and mounted with Antifade Reagents (ZSGB-BIO, Cat# ZLI-9557) for imaging and analysis.

For the quantification of MYC^+^, CD8^+^, and Tomato^+^ cells, we followed previously described methods 19. At least three sections from each HNSCC lesion were immunostained and analyzed. Tumor cells (> 150) and MYC^+^, CD8^+^, and Tomato^+^ cells in these tumor cell areas were counted manually in each section. The percentage of MYC^+^, CD8^+^, and Tomato^+^ cells were calculated by dividing those cells with tumor cells and were averaged from the sections.

### Quantitative real-time reverse transcription PCR (qRT-PCR)

Total RNA was prepared using the TRIzol reagent (Thermo Fisher Scientific, Cat# 15596026), and 500 ng of RNA was reverse transcribed to cDNA using a reverse transcription kit (Takara, Dalian, China; Cat# RR036A). The cDNA was then quantified using a SYBRGreen kit (Roche, Basel, Switzerland; Cat# 04913914001). *GAPDH* (encoding glyceraldehyde-3-phosphate dehydrogenase) was detected as the internal control. The qRT-PCR primers are listed in Table S1.

### Western blot

Cells were lysed using radioimmunoprecipitation assay (RIPA) buffer (Solarbio, Cat# R0010) and added with a cocktail of protease inhibitors and phosphatase inhibitors (Huaxingbio, Chaohu, China; Cat# HX1864). Total proteins (30 μg) were separated using SDS polyacrylamide gel electrophoresis (SDS-PAGE) and then transferred onto polyvinylidene fluoride membranes. The membranes were blocked with 5% skim milk for 1 h and then incubated with primary antibodies. The primary antibodies used in this study were: anti-MYC (Cell Signaling Technology; Cat# 18583; 1:1000), anti-B lymphoma Mo-MLV insertion region 1 homolog (BMI1) (Cell Signaling Technology; Cat# 6964; 1:1000), anti-SRY-box transcription factor 2 (SOX2) (Cell Signaling Technology; Cat# 14962; 1:1000), anti‑ALDH1 (Cell Signaling Technology; Cat# 54135; 1:1000), anti-phospho-Histone H2A.X (Cell Signaling Technology; Cat# 9718; 1:1000), anti-p-IRF3 (Cell Signaling Technology; Cat# 37829; 1:1000), anti-GAPDH (ZSGB-BIO; Cat# TA-08; 1:1000), and anti-Histone H3 (ABclonal, Wuhan, China; Cat# A2348; 1:1000). The membranes were then stained with the appropriate secondary antibodies. The signals were detected using a Clarity Western ECL kit (Thermo Fisher Scientific; Cat# 34577).

### Cytosolic dsDNA Staining

Following treatment, HN6 and SCC15 cells were incubated with culture medium containing PicoGreen (a dsDNA stain, 200-fold dilution, Thermo Fisher Scientific; Cat# P11496) and MitoTracker (mitochondrial dsDNA stain, 500 nM, Thermo Fisher Scientific; Cat# M7512). At 1 h after incubation, the cells were fixed using 4% paraformaldehyde for 10 min. Cells were then washed three times with phosphate‑buffered saline (PBS) and stained with DAPI (ZSGB-BIO; Cat# ZLI-9557). Staining was imaged and assessed using a Leica SP5X laser scanning confocal microscope (Leica, Wetzlar, Germany).

### Comet assays

Single cell gel electrophoresis (SCGE) comet assays were performed using an SCGE assay Kit (Enzo Life Sciences, Farmingdale, NY, USA; Cat# ADI-900-166). Following treatment, the cells were mixed with low melting point agarose at a volume ratio of 1:50, and 75 μL aliquots were loaded onto pre-warmed slides. The slides were incubated in pre-chilled lysis solution for 1 h and then in pre-chilled alkaline solution for 30 min. Electrophoresis was run at 25 V in Tris/Borate/EDTA (TBE) buffer for 15 min. Comets were stained using CYGREEN dye (Enzo Life Sciences) for 30 min and imaged. At least 100 individual cells per sample were evaluated in duplicate using the CASP Version 1.2.2 analysis tool (СASPlab, Wroclaw, Poland).

### Statistical analysis

SPSS 21.0 (IBM Corp., Armonk, NY, USA) was used for the statistical analysis. All *in vitro* experiments were repeated at least two times, and the* in vivo* experiments were repeated at least once. Student's* t* test was used to analyze the data between two groups. The differences among multiple groups were evaluated using one-way analysis of variance (ANOVA). A χ^2^ test and Cochran-Armitage test were used to analyze the HNSCC invasion data. *P* < 0.05 was considered statistically significant.

## Results

### MYC is high expressed in cisplatin-resistant HNSCC models

**Figure 1:**
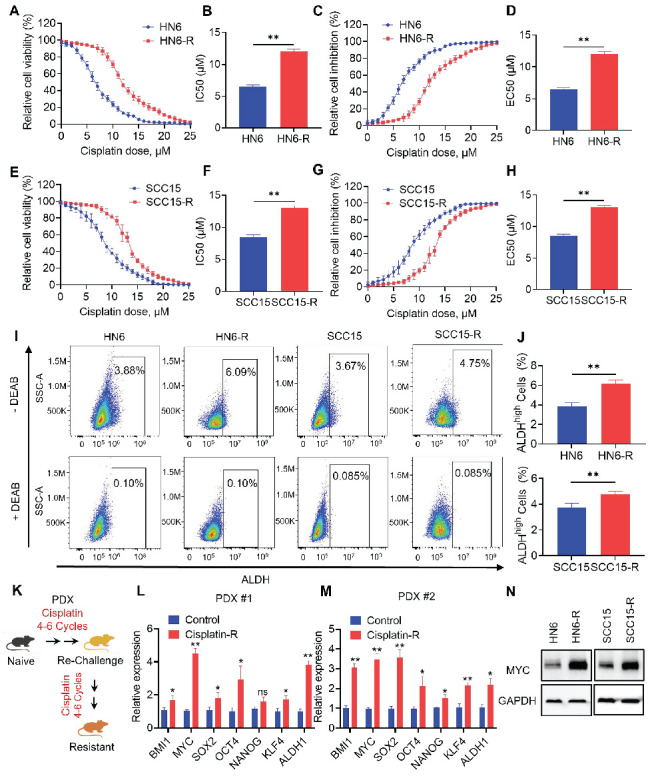
** MYC is highly expressed in cisplatin-resistant HNSCC models.** (A-B) CCK-8 assay analysis of the IC50 of cisplatin in parental and cisplatin-resistant HN6 cells. (C-D) CCK-8 assay analysis of the EC50 of cisplatin in parental and cisplatin-resistant HN6 cells. (E-F) CCK-8 assay analysis of the IC50 of cisplatin in parental and cisplatin-resistant SCC15 cells. (G-H) CCK-8 assay analysis of the EC50 of cisplatin in parental and cisplatin-resistant SCC15 cells. (I-J) Representative FACS plots of ALDH^high^ cells in HN6 and SCC15 cells with or without cisplatin resistance. Means ± SD are shown. **P < 0.01 using an unpaired Student's *t* test. A specific inhibitor of ALDH, diethylaminobenzaldehyde (DEAB), was used to control for background fluorescence. (K) Schematic diagrams of acquired cisplatin resistance PDX model generation. (L-M) qRT-PCR analysis of HNSCC CSC-characteristic genes in two paired PDX models. Means ± SD are shown. *P < 0.05 and **P < 0.01 using an unpaired Student's *t* test. (N) Western blot analysis of MYC protein levels in HN6 and SCC15 cells with or without cisplatin resistance. GAPDH was used as the internal control.

To explore the role of CSCs in cisplatin-resistant, we established cisplatin‑resistant HN6 (HN6-R) and cisplatin-resistant SCC15 (SCC15-R) cells. The IC50 and EC50 of the resistant cells were increased than those of the parental cells (Figure 1A, 1B, 1C, 1D, 1E, 1F,1G and 1H). Compared with that of the parental cells, the proliferation of HN6-R and SCC15-R cells was similar under cisplatin (3 µM) treatment (Figure S1A and S1B). Furthermore, TUNEL assays showed that HN6-R and SCC15-R cells were resistant to cisplatin‑induced apoptosis (Figure S1C, S1D, S1E and S1F). Previous studies have successfully used ALDH activity to isolate CSCs from human HNSCC cell lines and primary tumor tissues 15, 17, 18, 23. Using an ALDEFLUOR kit, we detected that ALDH^high^ CSC‑like cells were enriched among HN6-R and SCC15-R cells (Figure 1I and 1J).

To simulate acquired cisplatin resistance *in vivo*, we adopted an analogous approach of repeated chemotherapy cycles in human PDX models of HNSCC to select populations of tumors that were resistant to cisplatin (Figure 1K). We established two PDX models with different genotypes for resistance to cisplatin, as previously described 21. Then, we focused on analyzing potential cancer stemness markers of acquired cisplatin resistance. The mRNA expression of CSC-related genes, including *BMI1*, *MYC*, *SOX2*, *OCT4*, *NANOG*, *KLF4*, and *ALDH1* were analyzed in two paired PDX models with or without cisplatin resistance. qRT-PCR showed that *MYC* was one of the mostly high expressed genes in cisplatin resistant PDX models (Figure 1L and 1M). The MYC protein level was also increased in HN6-R and SCC15-R compared with that in the controls (Figure 1N). Thus, we chose MYC for further investigation.

### MYC inhibition reduces the stemness of HNSCC cells

**Figure 2:**
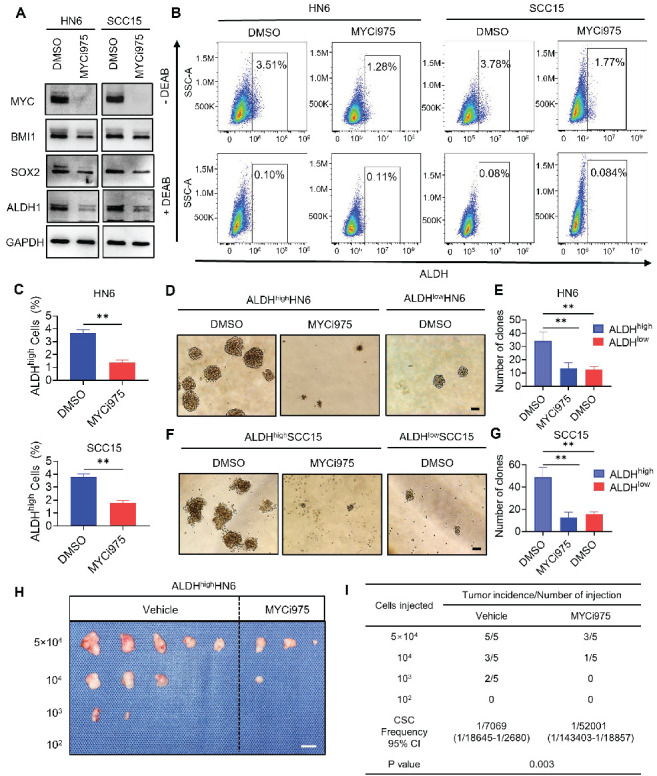
** MYCi975 suppresses cancer stemness in HNSCC cells.** (A) Western blot analysis showing the inhibition of MYC, BMI1, SOX2, and ALDH1 after MYCi975 treatment. GAPDH was used as the internal control. (B-C) Representative FACS plots of ALDH^high^ cells in HN6 and SCC15 cells after MYCi975 treatment. Means ± SD are shown. **P < 0.01 using an unpaired Student's *t* test. A specific inhibitor of ALDH, diethylaminobenzaldehyde (DEAB), was used to control for background fluorescence. (D-G) Images and numbers of tumorspheres formed by ALDH^high^ and ALDH^low^ HN6 and SCC15 cells, with or without MYCi975 treatment. Scale bar: 100 μm. Means ± SD are shown. **P < 0.01 using an unpaired Student's *t* test. (H-I) Extreme limiting dilution analysis (ELDA) of ALDH^high^ CSC‑like cells from HN6 cells treated with or without MYCi975 *in vivo* (n = 5). The frequency of allograft formation is displayed for each cell dose injected. ELDA software was used to analyze the data. Scale bar: 1 cm.

To investigate whether MYC-targeted therapy could eliminate CSCs in HNSCC, we took advantage of the specific inhibitor of MYC, MYCi975, which has been shown to effectively inhibit MYC 24-26. Inhibition of MYC by MYCi975 in HN6 and SCC15 parental and cisplatin-resistant cells was confirmed by Western blot (Figure 2A and S2A). We also found that levels of CSC-related proteins, BMI1, SOX2, and ALDH1, were reduced under MYCi975 treatment. *MYC* KD using an shRNA produced similar results (Figure S3A). FACS analysis showed that MYCi975 or shMYC treatment reduced the proportion of ALDH^high^ CSC-like cells among HN6 and SCC15 cells (Figure 2B, 2C, S3B, and S3C). Tumorsphere formation assays showed that MYCi975 inhibited the size and number of the ALDH^high^ CSC-like cell spheres (Figure 2D, 2E, 2F and 2G). Moreover, *MYC* KD using an shRNA in ALDH^high^ CSC‑like cells led to similar results (Figure S3D, S3E, S3F and S3G). In addition, *in vivo* limiting dilution tumorigenicity assays showed that MYCi975 inhibited the tumorigenic potential of ALDH^high^ HN6 cells in nude mice (Figure 2H and 2I). To further confirm whether MYC controls the tumorigenic potential of CSCs, we isolated CSCs from the HNSCC PDX models using EpCAM^+^ALDH^high^ markers, as described previously 15 (Figure S4A). *In vivo* limiting dilution tumorigenicity assays showed that MYCi975 inhibited the tumorigenic potential of EpCAM^+^ALDH^high^ CSCs in nude mice (Figure S4B and S4C). These findings suggested that MYC regulates the pro-tumorigenic potential and self-renewal of CSCs.

### MYC inhibition suppresses the tumorigenic potential and metastasis of CSCs, thereby overcoming cisplatin resistance of HNSCC

**Figure 3:**
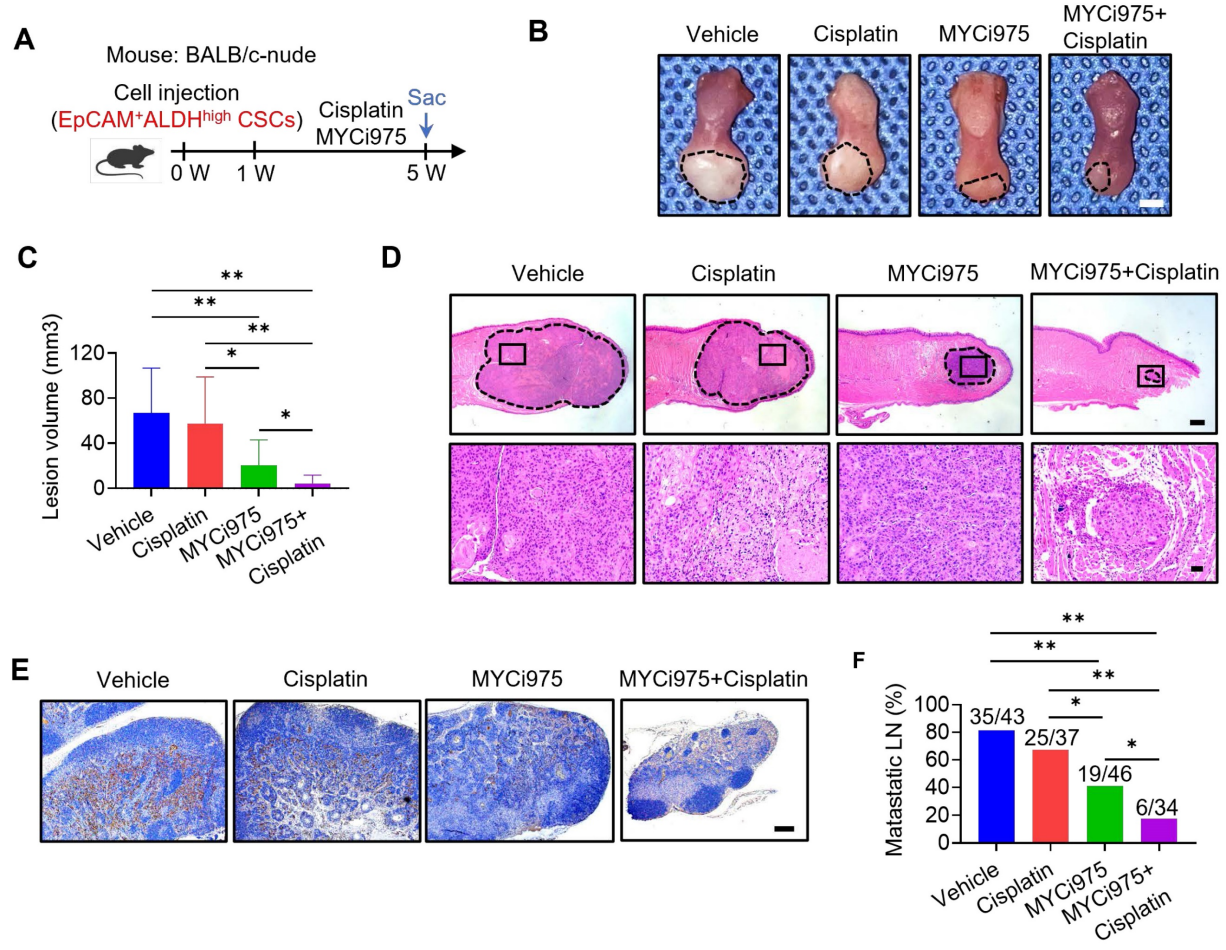
** MYCi975 suppresses the tumorigenic potential and metastasis of CSCs, and overcomes cisplatin resistance of HNSCC.** (A) Schematic diagram of the time points of MYCi975 and cisplatin treatment and sacrifice (Sac) of mice injected with EpCAM^+^ALDH^high^ CSCs from HNSCC PDX tissue. (B) Representative image of nude mice orthotopic tumors in the tongue from the mice injected with EpCAM^+^ALDH^high^ CSCs in the different treatment groups. The lesion areas are circled using a white dotted line. Scale bar, 2 mm. (C) Quantification of the tumor volume of nude mice in the different treatment groups. The values are means ± SDs from the pool of two independent experiments. n = 10. *P < 0.05 and **P < 0.01 using one-way ANOVA. (D) Representative H&E staining of nude mice orthotopic tumors. Scale bar, 500 μm. Enlarged images are shown in the lower panels. Scale bar, 50 μm. (E) Immunostaining of metastatic cells in cervical lymph nodes using anti-PCK antibodies. Scale bar, 200 μm. (F) Percentage of metastatic lymph nodes from mice. The number of metastatic lymph nodes in each group is indicated in the figure. *P < 0.05 and **P < 0.01 using a χ^2^ test.

HNSCC tumor cells, most likely CSCs, frequently metastasize to, and grow in, cervical lymph nodes. To explore whether MYC inhibition suppresses CSCs and their progeny's ability to develop HNSCC and metastasize, we isolated EpCAM^+^ALDH^high^ CSCs from the HNSCC PDX model and inoculated them into mice tongues to established an orthotopic HNSCC model. Mice were treated with control vehicle, cisplatin, MYCi975, or MYCi975 plus cisplatin for 4 weeks (Figure 3A). The reduction in MYC level induced by MYCi975 in tumors was confirmed by immunostaining (Figure S5A and S5B) and Western blot (Figure S5C). Treatment with MYCi975 alone reduced the orthotopic tumor growth, whereas cisplatin did not show inhibition compared with the control vehicle group. The addition of MYCi975 to cisplatin further enhanced the inhibitory effects compared with MYCi975 alone (Figure 3B, 3C and 3D). Cervical lymph node metastasis is a key prognostic factor for patients with HNSCC. To accurately detect whether the treatment inhibited HNSCC lymph node metastasis, the cervical lymph nodes of mice were immunostained with anti-PCK antibodies, which revealed that MYCi975 alone, or MYCi975 plus cisplatin, significantly reduced lymph node metastasis, whereas cisplatin alone could not suppress lymph node metastasis compared with the control vehicle group (Figure 3E and 3F). These findings suggested that CSCs were resistant to cisplatin, and MYC inhibition suppressed CSCs and lymph node metastasis of HNSCC.

**Figure 4:**
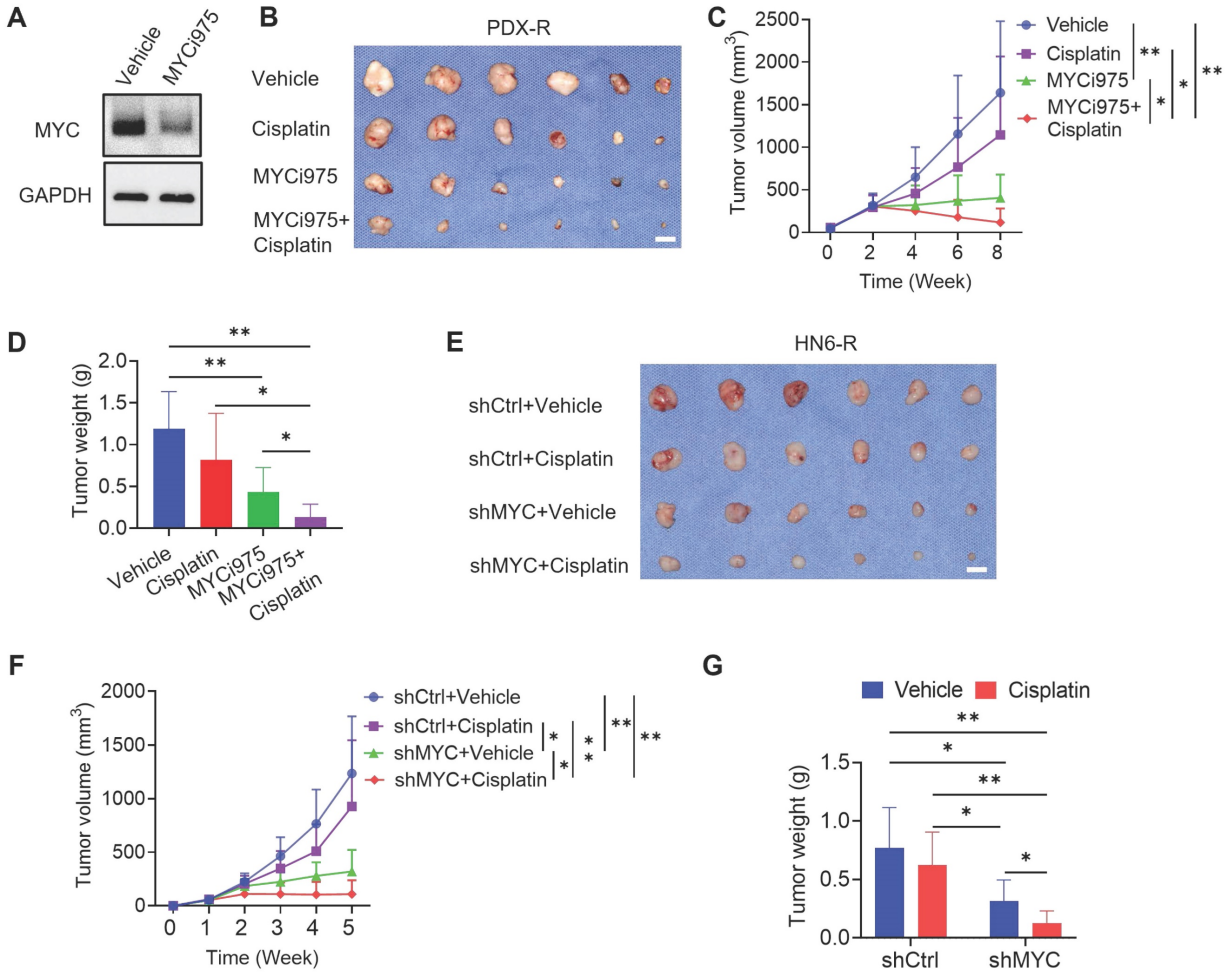
** MYCi975 overcomes cisplatin resistance of HNSCC.** (A) Western blot showing the reduction in MYC levels in PDX-R tumor tissues after MYCi975 treatment. GAPDH was used as the internal control. (B) Representative image of tumor samples harvested from cisplatin-resistant PDX mice. (C) Tumor volume growth curve of cisplatin-resistant PDX mice. *P < 0.05 and **P < 0.01 using one-way ANOVA. (D) Tumor weights of cisplatin-resistant PDX mice after 6 weeks of treatment. *P < 0.05 and **P < 0.01 using one-way ANOVA. (E) Representative image of tumor samples harvested from nude mice with injection of HN6-R cells. (F) Tumor volume growth curve of subcutaneous tumor models in nude mice with injection of HN6-R cells. *P < 0.05 and **P < 0.01 using one-way ANOVA. (G) Tumor weights of subcutaneous tumor models in nude mice with injection of HN6-R cells after 4 weeks of treatment. *P < 0.05 and **P < 0.01 using one-way ANOVA.

To further confirm these results, the cisplatin-resistant PDX model was treated with control vehicle, cisplatin, MYCi975, or MYCi975 plus cisplatin for 4 weeks. The reduction in the MYC level induced by MYCi975 in tumors was confirmed by Western blot (Figure 4A). The cisplatin-resistant PDX model showed no response to cisplatin treatment, whereas MYCi975 alone or MYCi975 plus cisplatin significantly inhibited tumor growth in terms of volume and weight (Figure 4B, 4C and 4D), suggesting that MYC inhibition overcame cisplatin resistance of HNSCC *in vivo*.

Meanwhile, to investigate whether *MYC* KD could overcome cisplatin resistance of HNSCC, we used shMYC to knockdown *MYC* in HN6-R cells. HN6-R cells transfected by shMYC or shCtrl were injected into the subcutaneous dorsal region of nude mice. One week after injection, the mice were divided in to difference groups. Mice injected with HN6-R cells transfected by shCtrl showed no response to cisplatin. shMYC-mediated KD of *MYC* significantly reduced the weight and volume of HN6-R-derived tumors in nude mice compared with that achieved by shCtrl and overcome cisplatin resistance (Figure 4E, 4F and 4G).

### MYC inhibition plus cisplatin eliminates CSCs in vivo, potently inhibiting HNSCC

Previously, we established a 4NQO-induced HNSCC *Bmi1^CreER^; Rosa^tdTomato^* mouse model, which allows us to perform the lineage tracing of CSCs in an *in vivo* unperturbed environment using tamoxifen-induced Cre-mediated recombination 19. To explore whether targeting MYC could suppress CSCs *in vivo* and augmented cisplatin therapy, we treated *Bmi1^CreER^; Rosa^tdTomato^* mice with 4NQO in their drinking water for 16 weeks and then replaced it with normal drinking water.

**Figure 5:**
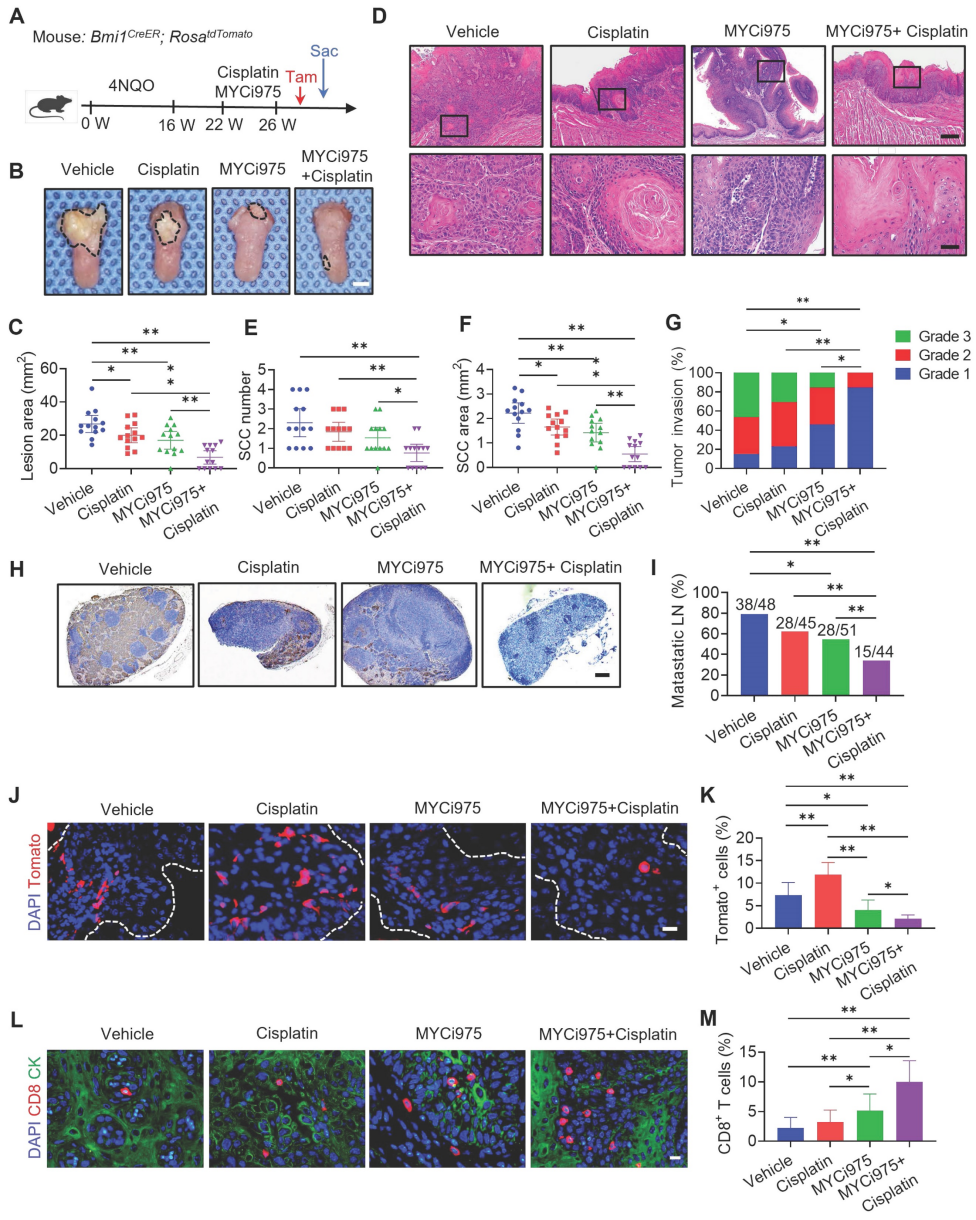
** MYCi975 plus cisplatin eliminates CSCs *in vivo*, potently inhibits HNSCC.** (A) Schematic diagrams showing the treatment and lineage tracing of primary HNSCC in *Bmi1^CreER^; Rosa^tdTomato^* mice. Tamoxifen (Tam) was administered 1 day before sacrificing (Sac) the mice to label BMI1+ CSCs. (B) Representative image of tongue visible lesions in the different treatment groups. Black dashed lines denote the lesion areas. Scale bar, 2 mm. (C) Quantification of the HNSCC lesion area from mice treated as indicated. The values are means ± SDs from the pool of two independent experiments. n = 13. (D) Representative H&E staining of HNSCC from mice treated as indicated. Scale bar, 200 μm. Enlarged images are shown in the lower panels. Scale bar, 40 μm. (E-F) Quantification of HNSCC tumor number and area from mice treated as indicated. The values are means ± SDs from the pool of two independent experiments. n = 13. *P < 0.05 and **P < 0.01 using one-way ANOVA. (G) Quantification of HNSCC invasion grades from mice treated as indicated. The data were pooled from two independent experiments. n = 13. *P < 0.05 and **P < 0.01 using the Cochran-Armitage test. (H) Immunostaining of metastatic cells in cervical lymph nodes using anti-PCK antibodies. Scale bar, 200 μm. (I) Percentage of metastatic lymph nodes from mice treated as indicated. The number of metastatic lymph nodes in each group is indicated in the figure. Data were pooled from two independent experiments. *P < 0.05 and **P < 0.01 using a χ^2^ test. (J) Representative images of Tomato^+^ BMI1^+^ CSCs in HNSCC from mice treated as indicated. The white dashed lines denote the tumor-stromal junction. Scale bar, 20 μm. (K) Quantification of the percentage of Tomato^+^ cells in HNSCC from mice treated as indicated. The values are means ± SDs from the pool of two independent experiments. n = 13. *P < 0.05 and **P < 0.01 using one-way ANOVA. (L) Immunostaining images for CD8^+^ T cells from mice treated as indicated. Scale bar, 10 μm. (M) Quantification of CD8^+^ T cells in HNSCC from mice treated as indicated. The values are means ± SDs from the pool of two independent experiments. n = 13. *P < 0.05 and **P < 0.01 using one-way ANOVA.

At 22 weeks from the initial 4NQO treatment, tumor-bearing mice were treated with cisplatin, MYCi975, MYCi975 plus cisplatin, or control vehicle. A single dose of tamoxifen was administered 1 day before sacrificing the mice to label Tomato^+^ BMI1^+^ CSCs (Figure 5A). The reduction in MYC levels by MYCi975 in tumors was confirmed using immunostaining (Figure S6A and S6B). MYCi975 plus cisplatin significantly reduced more lesion surface areas compared with cisplatin or MYCi975 alone (Figure 5B and 5C). Histological analysis indicated that MYCi975 plus cisplatin significantly reduced HNSCC numbers, areas, and invasiveness compared with MYCi975 or cisplatin alone (Figure 5D, 5E, 5F and 5G). Moreover, the metastatic status of isolated cervical lymph nodes was compared between the groups. The results showed that MYCi975 plus cisplatin effectively eliminated the majority of lymph node metastasis of HNSCC, as determined by anti-PCK immunostaining (Figure 5H and 5I).

MYCi975 plus cisplatin efficiently inhibited HNSCC; therefore, we used *in vivo* labeling of BMI1^+^ CSCs to determine whether the combination could efficiently eliminate BMI1^+^ CSCs, which play a critical role in HNSCC chemoresistance and relapse 15. Consistent with previous studies 15, 19, cisplatin significantly enriched BMI1^+^ CSCs in HNSCC. In sharp contrast to cisplatin, *in vivo* labeling showed that MYCi975 reduced BMI1^+^ CSCs. MYCi975 plus cisplatin further enhanced CSC elimination compared with MYCi975 alone (Figure 5J and 5K).

We then investigated the effect of the combination treatment on antitumor T cell immunity. Immunostaining showed that MYCi975 significantly increased CD8^+^ T cell infiltration, which was further significantly increased by MYCi975 plus cisplatin treatment (Figure 5L and 5M).

To determine whether secondary cisplatin resistance in 4NQO-induced HNSCC responds to MYCi975, we set up a new experiment to establish 4NQO-induced HNSCC with secondary cisplatin resistance as previously described 15, 19. Administration of cisplatin for 4 weeks resulted in tumor stasis. Then, we extended our observation for additional 4 weeks. The results showed that cisplatin treatment discontinuation led to re-initiation of HNSCC growth. Next, mice with recurrent HNSCC were treated with cisplatin, MYCi975 or control vehicle for another 4 weeks (Figure S7A). Treatment with MYCi975 reduced the lesion surface areas, whereas cisplatin did not show inhibition compared with the control vehicle in recurrent HNSCC (Figure S7B and S7C). Histological analysis found that MYCi975 significantly reduced HNSCC numbers, areas, and invasiveness, while no such inhibitory effect was observed under cisplatin treatment (Figure S7D, S7E, S7F and S7G). Furthermore, immunostaining with anti-PCK antibodies revealed that MYCi975 inhibited lymph node metastasis effectively rather than cisplatin (Figure S7H and S7I). *In vivo* labeling of BMI1^+^ CSCs revealed that MYCi975 efficiently eliminated BMI1^+^ CSCs in recurrent HNSCC, while BMI1^+^ CSCs remained in recurrent HNSCC treated with cisplatin (Figure S7J and S7K). These results demonstrated that recurrent HNSCC after cisplatin therapy was resistant to further cisplatin treatment, while MYCi975 had a significant impact on recurrent and cisplatin secondary resistance HNSCC.

Toxicology studies were performed using C57BL/6J mice treated with MYCi975 or cisplatin. Histopathological analysis revealed that no tissue damage occurred in major organs (including the heart, liver, lung, and kidney) after MYCi975 or cisplatin administration (Figure S6C). In addition, the evaluation of routine blood and blood biochemical indicators also suggested that MYCi975 or cisplatin were well tolerated by the mice (Figures S6D and S6E).

### MYC knockout with cisplatin suppress HNSCC growth and metastasis

**Figure 6:**
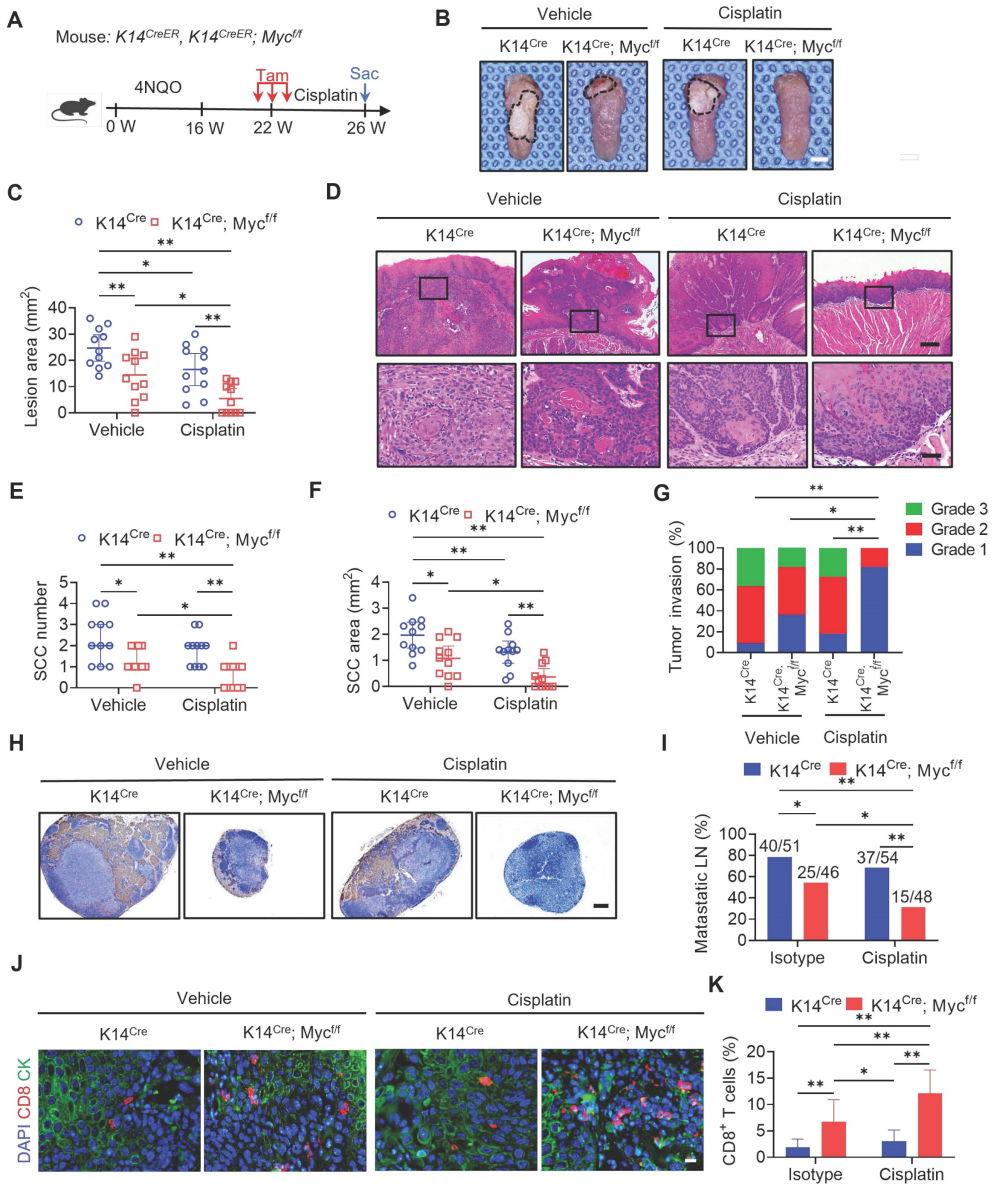
** Epithelial Deletion of MYC Collaborates with Cisplatin to Suppress HNSCC Growth and Metastasis.** (A) Experimental design for *MYC* knockout in tumor cells and Cisplatin treatment *in vivo*. Three administrations of Tam were given to tumor-bearing mice. Mice were randomly divided into four experimental groups (n = 11 per group from 2 independent experiments): *K14^Cre^* with control vehicle, *K14^Cre^* with cisplatin, *K14^Cre^; Myc^f/f^* with control vehicle, and *K14^Cre^; Myc^f/f^* with cisplatin. (B) Representative image of tongue visible lesions. Black dashed lines denote the lesion areas. Scale bar, 2 mm. (C) Quantification of the HNSCC lesion area from mice treated as indicated. The values are means ± SDs from the pool of two independent experiments. n = 11. (D) Representative H&E staining of HNSCC from mice treated as indicated. Scale bar, 200 μm. Enlarged images are shown in the lower panels. Scale bar, 40 μm. (E-F) Quantification of HNSCC tumor number and area from mice treated as indicated. The values are means ± SDs from the pool of two independent experiments. n = 11. *P < 0.05 and **P < 0.01 using one-way ANOVA. (G) Quantification of HNSCC invasion grades from mice treated as indicated. The data were pooled from two independent experiments. n = 11. *P < 0.05 and **P < 0.01 using the Cochran-Armitage test. (H) Immunostaining of metastatic cells in cervical lymph nodes using anti-PCK antibodies. Scale bar, 200 μm. (I) Percentage of metastatic lymph nodes from mice treated as indicated. The number of metastatic lymph nodes in each group is indicated in the figure. Data were pooled from two independent experiments. *P < 0.05 and **P < 0.01 using a χ^2^ test. (J) Immunostaining images for CD8^+^ T cells from mice treated as indicated. Scale bar, 10 μm. (K) Quantification of CD8^+^ T cells in HNSCC from mice treated as indicated. The values are means ± SDs from the pool of two independent experiments. n = 11. *P < 0.05 and **P < 0.01 using one-way ANOVA.

To further investigate whether targeting tumor cell-intrinsic MYC augmented cisplatin therapy, we crossed *Myc^flox/flox^* (*Myc^f/f^*) mice with keratin 14-Cre/ERT2 mice (*K14^CreER^*) to generate *K14^CreER^; Myc^flox/flox^* mice, in which epithelial *Myc* can be inducibly deleted by tamoxifen treatment. Three successive applications of tamoxifen were applied to both *K14^CreER^; Myc^flox/flox^* and the control *K14^CreER^* mice at 22 weeks after the initial 4NQO treatment (Figure 6A). *Myc* knockout (*Myc* KO) in mouse HNSCC was confirmed by immunostaining (Figure S8A and S8B). Treatment with cisplatin or *Myc* KO alone reduced the lesion areas; whereas, the addition of cisplatin to *Myc* KO further enhanced the inhibitory effects on the lesion areas compared with either single treatment (Figure 6B and 6C). Histological analysis revealed that *Myc* KO or cisplatin alone could reduce the numbers, areas, and invasive grades of HNSCC compared with those in the control vehicle group. *Myc* KO plus cisplatin significantly inhibited the numbers, areas, and invasive grades of HNSCC compared with *Myc* KO or cisplatin alone (Figure 6D, 6E, 6F and 6G). Moreover, *Myc* KO plus cisplatin also exhibited superior inhibitory effects on lymph node metastasis compared with *Myc* KO alone (Figure 6H and 6I). Immunostaining revealed that *Myc* KO alone, but not cisplatin, could induce CD8^+^ T cell infiltration in HNSCC, which was further increased in HNSCC treated with *Myc* KO plus cisplatin (Figure 6J and 6K).

### MYC inhibition plus cisplatin facilitates cellular DNA damage and increases the tumor cell-intrinsic immune response

**Figure 7:**
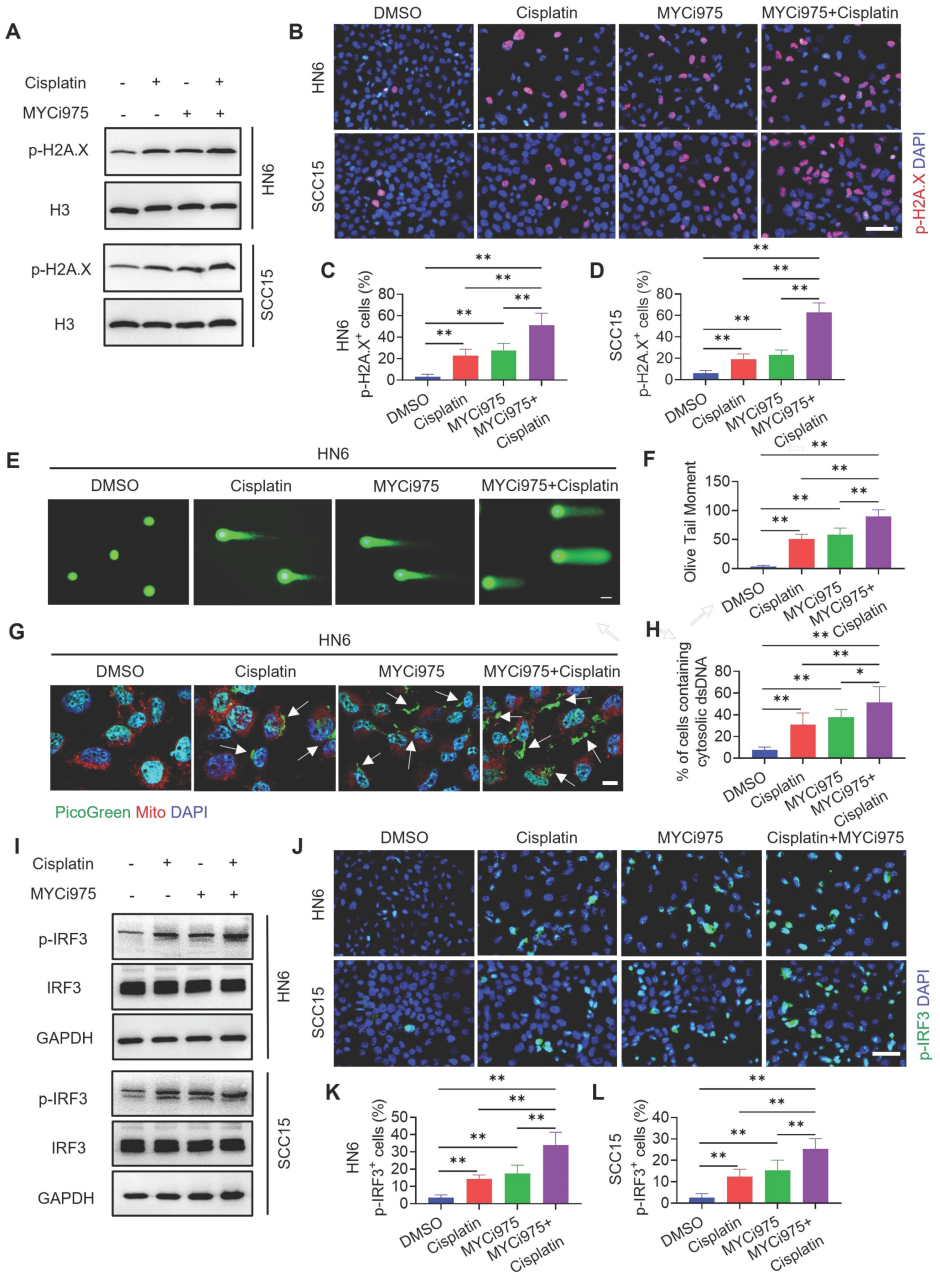
** MYCi975 plus cisplatin induces DNA damage and activates the cGAS‑STING-IRF3 signaling pathway.** (A) Western blot analysis of p-H2A.X in HN6 and SCC15 cell under different conditions as indicated. H3 was used as the internal control. (B-D) Immunostaining and quantification of p-H2A.X (red) in HN6 and SCC15 cells under different conditions as indicated. Nuclei were stained using DAPI (blue). Means ± SD are shown from three independent experiments. Scale bar, 50 μm. **P < 0.01 using one-way ANOVA. (E-F) Representative images and quantification of DNA Comet assays in HN6 cells treated under different conditions as indicated. More than 100 cells were analyzed per group. Means ± SD are shown. Scale bar, 100 μm. ** P < 0.01 using one‑way ANOVA. (G-H) Confocal microscopy images showing cytosolic DNA accumulation and quantification in HN6 cells under different conditions as indicated. Double‑stranded DNA (dsDNA) was stained using PicoGreen (green). Mitochondria and nuclei were stained using MitoTracker (Red) and DAPI (blue), respectively. The white arrows indicate cytosolic dsDNA. Scale bar, 10 μm. More than 100 cells were analyzed per group. Means ± SD are shown. ** P < 0.01 using one-way ANOVA. (I) Western blot analysis of the phosphorylation of IRF3 (p-IRF3) in HN6 and SCC15 cells under different conditions as indicated. GAPDH was used as the internal control. (J‑L) Immunostaining and quantification of p-IRF3 (green) in HN6 and SCC15 cells under different conditions as indicated. Nuclei are stained using DAPI (blue). Means ± SD are shown from three independent experiments. Scale bar, 50 μm. *P < 0.05 and ** P < 0.01 using one-way ANOVA.

Previous studies showed that MYC is associated with the DNA damage response and repair regulation 27-29. Our recent study showed that MYCi975 induced the DNA damage response 26. We hypothesized that MYCi975 plus cisplatin would enhance the DNA damage response. To test this hypothesis, we used Western blot and immunostaining to confirm that the level of p-H2A.X, a specific marker for DNA damage, was significantly increased in cells treated with MYCi975 plus cisplatin compared with that in cells treated with cisplatin or MYCi975 alone (Figure 7A, 7B, 7C and 7D). Furthermore, p-H2A.X was also increased in MYCi975-treated 4NQO induced‑HNSCC (Figure S9A and S9B). Comet assays showed that MYCi975 plus cisplatin treatment significantly increased the comet tail moment compared with that obtained using cisplatin or MYCi975 alone in HN6 and SCC15 cells (Figure 7E, 7F, S9C and S9D), which confirmed that damaged cellular DNA fragments were separated from intact DNA. DNA damage in the nucleus could bring about the cytosolic accumulation of DNA fragments. We stained live HNSCC cells with PicoGreen, a dsDNA-specific vital dye. It was reported that PicoGreen also stains mitochondrial DNA 30; therefore, mitochondrial DNA was visualized using MitoTracker staining to exclude its interference during the quantification of cytosolic dsDNA. Upon cisplatin or MYCi975 treatment, multiple PicoGreen staining areas were observed in the cytoplasm of HN6 and SCC15 cells, which did not overlap with the MitoTracker staining, indicating that MYCi975 induced the accumulation of cytosolic dsDNA. In addition, MYCi975 plus cisplatin enhanced the accumulation of cytosolic dsDNA (Figures 7G, 7H, S9E and S9F).

**Figure 8:**
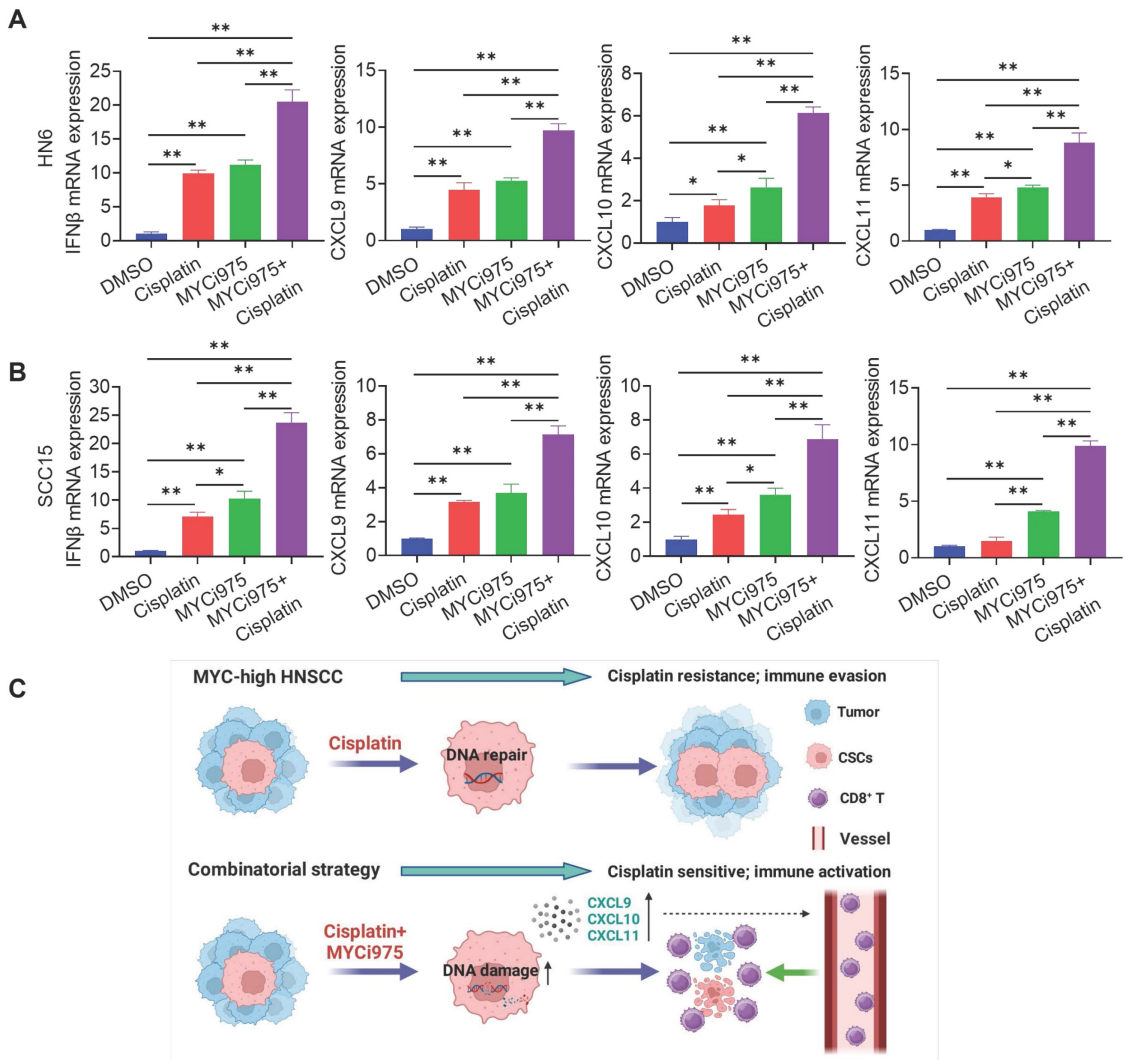
** MYCi975 plus cisplatin induces the expression of CD8^+^ T cell‑attracting chemokines in HNSCC cells.** (A) The mRNA expression levels of *IFNB*, *CXCL9*, *CXCL10*, and *CXCL11* in HN6 cells induced under different conditions as indicated. Means ± SD are shown. *P < 0.05 and **P < 0.01 using one-way ANOVA. (B) The mRNA expression levels of *IFNB*, *CXCL9*, *CXCL10*, and *CXCL11* in SCC15 cells induced under different conditions as indicated. Means ± SD are shown. *P < 0.05 and **P < 0.01 using one-way ANOVA. (C) A diagram of the mechanism by which MYCi975 plus cisplatin eliminate CSCs and activate antitumor immunity to overcome cisplatin resistance.

DNA damage can activate a tumor immune response 19, 31-33. The accumulation of cytosolic DNA can activate the cyclic guanosine monophosphate (GMP)-AMP synthase-stimulator of interferon (IFN) gene (cGAS-STING) signaling axis by the sequential phosphorylation of IRF3. Then, p-IRF3 dimerizes and translocates to the nucleus to trigger the expression of IFN and IFN-regulated chemokines 34, including C-X-C motif chemokine ligand (CXCL)9, CXCL10, and CXCL11^35^. Western blot (Figure 7I) and immunostaining (Figure 7J, 7K and 7L) analysis showed that MYCi975 plus cisplatin enhanced the levels of p-IRF3 in HN6 and SCC15 cells. Consequently, MYCi975 plus cisplatin increased the mRNA expression levels of *IFNB*, *CXCL9*, *CXCL10*, and *CXCL11* in HN6 and SCC15 cells (Figure 8A and 8B). We also observed that the p-IRF3 level was significantly increased in 4NQO induced-HNSCC upon MYCi975 plus cisplatin treatment, confirming that MYCi975 plus cisplatin enhanced the activation the cGAS-STING-IRF3 pathway *in vivo.* (Figures S9G and S9H).

## Discussion

CSCs have been demonstrated to play a critical role in tumor development, progression, and metastasis 19, 36. Traditional chemotherapies, such as cisplatin, are effective at abolishing actively proliferating tumor cells, but are usually not effective to eradicate CSCs, which leads to CSC enrichment and tumor recurrence 15, 16, 36. In this study, we demonstrated that MYC inhibition could overcome cisplatin resistance in HNSCC. The combination treatment of MYCi975 plus cisplatin not only potently inhibited HNSCC invasive growth, but also significantly reduced lymph node metastasis. Mechanistically, while MYC inhibition destroyed CSCs, it also augmented the ability of cisplatin to kill non-stem tumor cells in HNSCC by enhancing the DNA damage response, which activated antitumor immunity by recruiting CD8^+^ T cells through chemokines (Figures 8C). Importantly, we showed that MYC plays a critical role in CSC tumorigenic potential and the survival of HNSCC, highlighting that MYC might be a new therapeutic target for the effective elimination of human HNSCC CSCs.

Cisplatin remains the most effective first-line agent for recurrent and metastatic HNSCC 7. Although cisplatin improves important organ preservation and enhances the quality of life of patients with HNSCC 37, patients rapidly develop chemoresistance and are able to regrow and regenerate tumors locally and/or at distant sites. The overall survival of patients with head and neck cancer has not improved significantly for the last 40 years 1, despite the extensive use of platinum-based therapies for the last three decades. Based on the CSC hypothesis, CSCs might be associated with tumor development, metastasis, recurrence, and chemotherapy resistance because of their self-renewing capacity 38. Understanding the epigenetic mechanisms that regulate the cancer stemness will help to develop new targeting strategies to eliminate the CSCs.

Our results showed that MYC was highly expressed in cisplatin-resistant PDX models. Growing evidence indicates that MYC regulates tumorigenesis through a variety of mechanisms, and is functionally involved in up to 70% of all human cancers 20. Multiple studies demonstrated that MYC plays an important role in tumorigenesis, and is regarded as an attractive target for cancer therapy 39-45. Recently, we discovered that MYC was associated with lymph node metastasis and poor prognosis in HNSCC 26. In the present study, *in vitro* data showed that MYC inhibition suppressed tumorsphere formation and the tumorigenic potential of CSCs in HNSCC, indicating that MYC plays a critical role in CSC tumorigenic potential. Furthermore, our orthotopic tumor model suggested that MYCi975 could effectively inhibit CSCs and their progeny's ability to develop HNSCC *in vivo*. MYCi975 not only inhibited MYC, but also disrupted well-documented CSC-related proteins, BMI1, SOX2, and ALDH1. Previous studies identified that BMI1^+^ CSCs were resistant to cisplatin treatment in HNSCC 15, 19, 46, and SOX2 is another important transcription factor regulating CSC properties 47. Using *in vivo* lineage tracing, we identified that cisplatin enriched BMI1^+^ CSCs, while MYCi975 could effectively eliminate cisplatin‑enriched CSCs. These findings indicated that targeting MYC is a good strategy to suppresses cancer stemness and MYCi975 might be an effective therapeutic approach to overcome drug resistance in cancer therapy.

In our study, other CSC-associated genes also raise in the cisplatin-resistant PDX models. Our previous study showed that the BMI1 inhibitor could effectively inhibit HNSCC growth, and prevent metastasis and relapse 19. However, we also observed that MYC was elevated after BMI1 inhibition in another study. Thus, the use of BMI1 inhibitors is not appropriate for the treatment of cisplatin resistance HNSCC with MYC elevation. For other CSC-associated proteins, such as SOX2, OCT4, and KLF4, there are still no appropriate drugs to use. For NANOG and ALDH1 inhibitors, such as Amcasertib and Disulfiram 48, 49, our preliminary data showed that their anti-tumor effect in HNSCC was not as good as MYCi975.

HNSCC frequently metastasizes to cervical lymph nodes, and the poor prognosis of patients with HNSCC is closely associated with lymph node metastasis 50, 51. A previous study suggested that CSCs might be associated with tumor metastasis 15. In this study, we demonstrated that MYCi975 effectively inhibited tumor growth and metastasis by eliminating CSCs. Although cisplatin could reduce the tumor volume, it could not eliminate CSCs and suppress metastasis 52. Using orthotopic tumor and 4NQO-induced mouse models, we identified that cisplatin alone could not suppress lymph node metastasis of HNSCC, but MYCi975 effectively suppressed lymph node metastasis and this suppression was enhanced using the combination therapy.

Furthermore, we found that MYCi975 could overcome cisplatin resistance and demonstrated that MYCi975 combined with cisplatin potently inhibited HNSCC. A previous study provided evidence that cisplatin treatment mainly killed proliferating cells, rather than CSCs 15. Although the use of the MYC inhibitor impaired HNSCC growth and metastasis, it was not as effective as the combination therapy. We speculated that there are several potential reasons. Although MYCi975 could ablate CSCs with high MYC expression, the tumor cells with low MYC expression were able to proliferate and sustain the tumor bulk until they were worn out. It was also possible that non-stem tumor cells might have evolved and dedifferentiated into CSCs in the tumor microenvironment. Targeting MYC might promote tumor cells to dedifferentiate into other different CSCs. In fact, a previous study suggested that cytokines or growth factors could activate oncogenic signaling pathways that might be able to reprogram or dedifferentiate non-stem tumor cells into CSCs 53.

Immune surveillance is critical to prevent tumor development and progression. HNSCC tumor cells, most likely CSCs, likely have some unique mechanisms to evade immune surveillance 54. Recently, we showed that MYC inhibition leads to cellular apoptosis and the DNA damage response 26. In this study, while MYCi975 suppressed CSCs in HNSCC, it also activated antitumor immunity by recruiting CD8^+^ T cells through the cGAS-STING signaling pathway. Moreover, we demonstrated that the combination therapy enriched this effect. Initially, we showed that the extent of DNA damage was increased by the combination therapy, compared with that induced by MYCi975 or cisplatin alone, using p-H2A.X and comet assays. Then, consistent with DNA damage, we confirmed the accumulation of cytosolic dsDNA, which subsequently activated the phosphorylation of IRF3 to induce the expression of type I IFN chemokines (CXCL9, CXCL10, and CXCL11) and activated CD8^+^ T cell recruitment. This also explained the reason for the effective treatment by MYCi975 and cisplatin combination therapy. Previous studies demonstrated that MYC inhibition modulates the tumor immune microenvironment and enhances anti-PD1 immunotherapy 24. The close relationship between cGAS-STING-IRF3 pathway and immunotherapy was also well documented 19, 31, 34, suggesting that MYCi975 could be also used to enhance the sensitivity of HNSCC to immunotherapy in the future.

## Conclusions

The results demonstrated that MYCi975 could eliminate CSCs, prevent metastasis, activate the intrinsic immune responses, and overcome cisplatin resistance in HNSCC. We propose MYCi975 combined with cisplatin as a potential therapeutic option for clinical trials to treat HNSCC.

## Supplementary Material

Supplementary figures.

## Abbreviations

HNSCC: head and neck squamous cell carcinoma
CSC: cancer stem cell
PDX: patient-derived xenograft
qRT-PCR: quantitative real-time reverse transcription PCR
SD: standard deviation
TUNEL: terminal deoxynulceotidyl transferase nick-end-labeling
4NQO: 4-nitroquinoline 1-oxide
MYC: MYC proto-oncogene, BHLH transcription factor
ALDH: alcohol dehydrogenase
BMI-1: B Lymphoma Mo-MLV insertion region 1 homolog
CCK8: cell counting kit 8
DAPI: 4, 6-diamidino-2-phenylindole
FACS: fluorescence activated cell sorting
GAPDH: glyceraldehyde-3-phosphate dehydrogenase
KLF4: Kruppel like factor 4
OCT4: octamer-binding protein 4
SOX2: SRY-box transcription factor 2
CXCL: C-X-C motif chemokine ligand
IFN: interferon
IRF3: interferon regulatory factor 3
cGAS-STING: cyclic guanosine monophosphate-adenosine monophosphate synthase-stimulator of interferon gene
