# Supplementary material for: Pharmacological inhibition of MYC to mitigate chemoresistance in preclinical models of squamous cell carcinoma
Source: Theranostics. 2024 Jan 1;14(2):622–39. doi: 10.7150/thno.88759 (PMC10758066; doi:10.7150/thno.88759)
Supplement: Supplementary file 1 — Supplementary figures. [file thnov14p0622s1.pdf]

Figure S1. Related to Figure 1

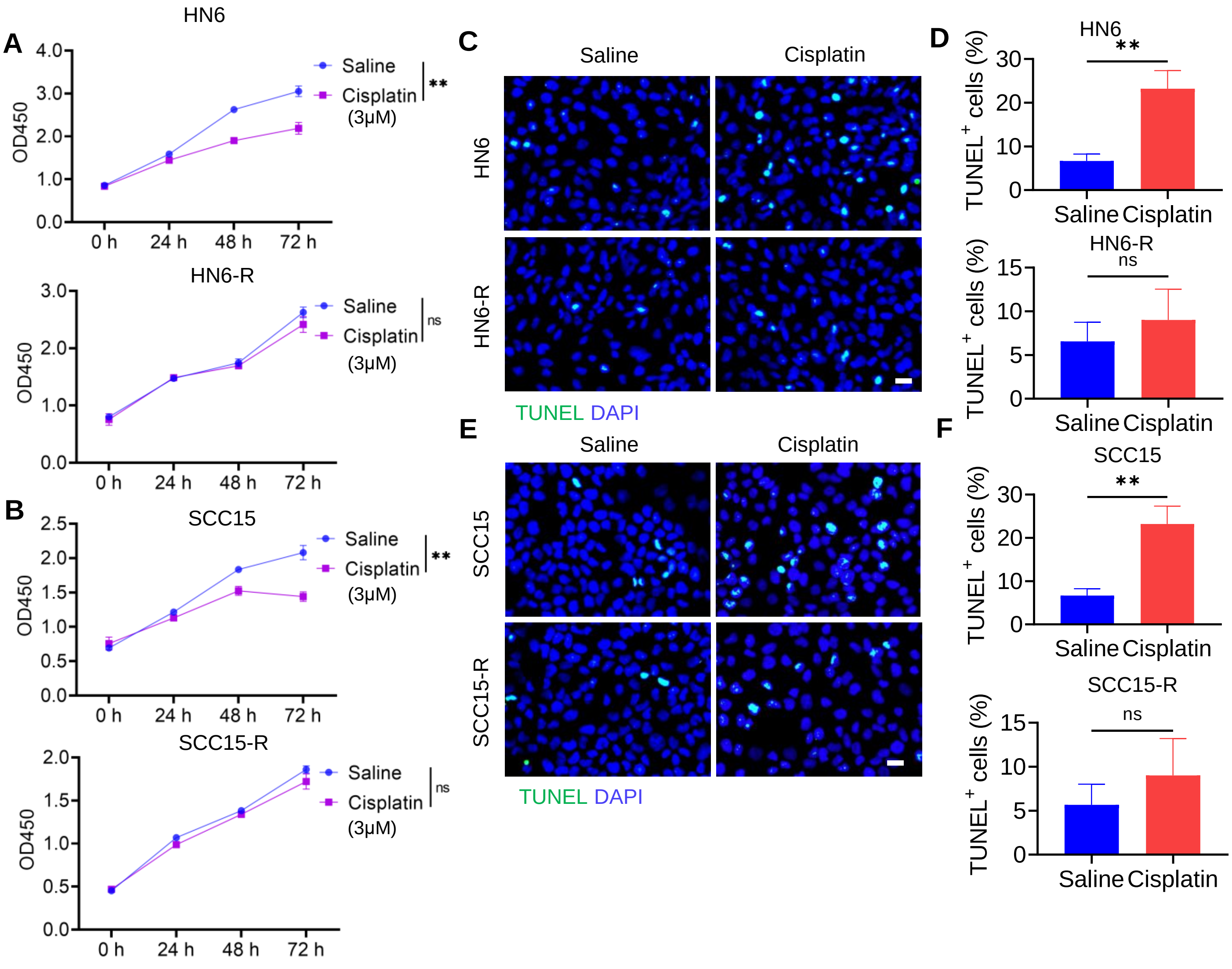

Figure S2. Related to Figure 2

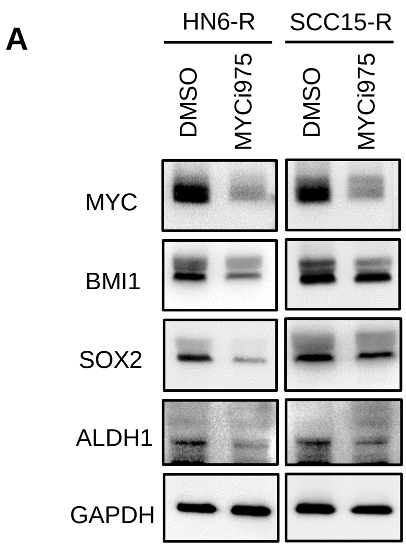

Figure S3. Related to Figure 2

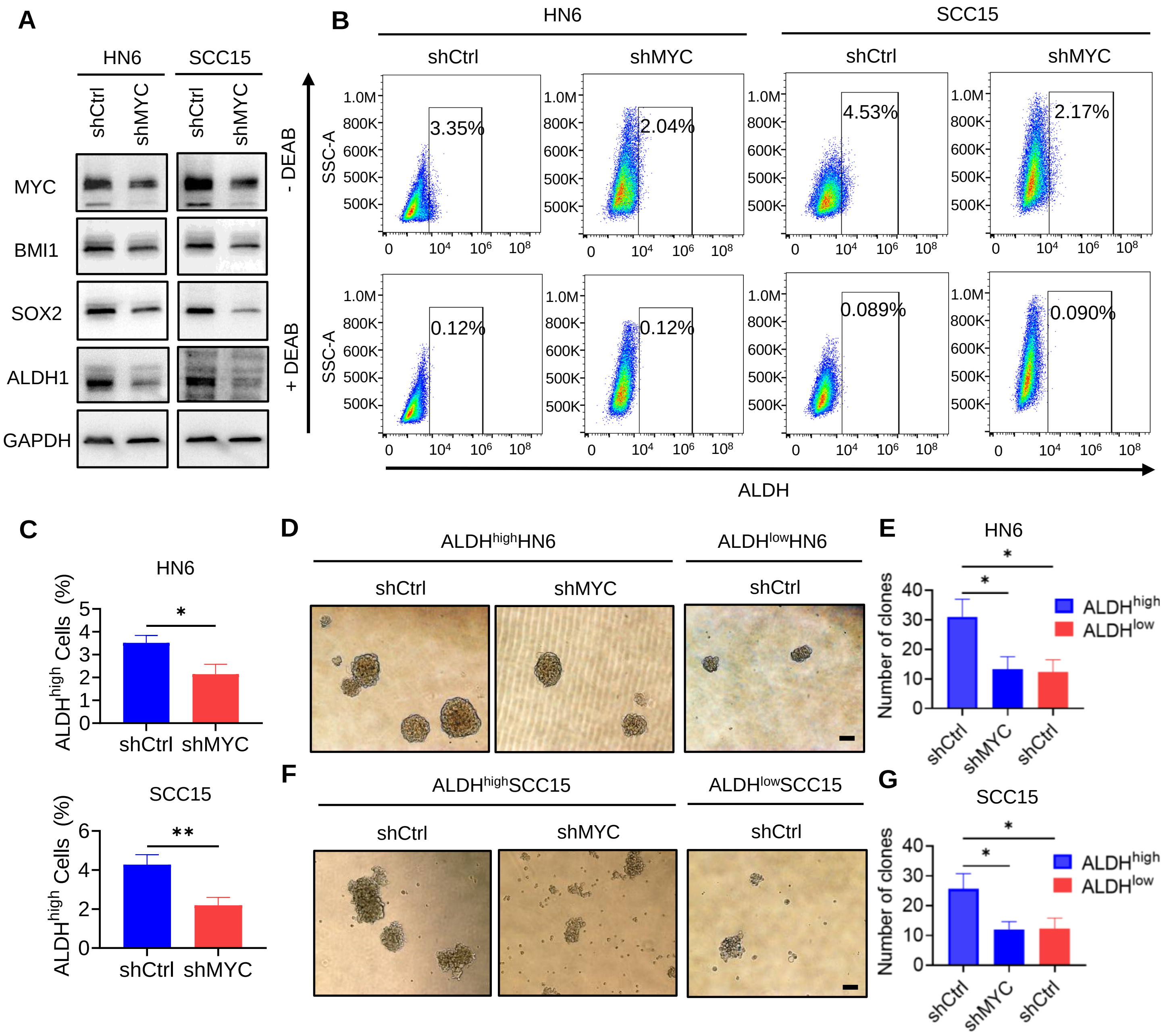

Figure S4. Related to Figure 2

A

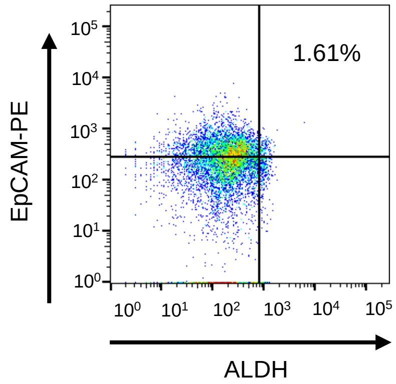

B

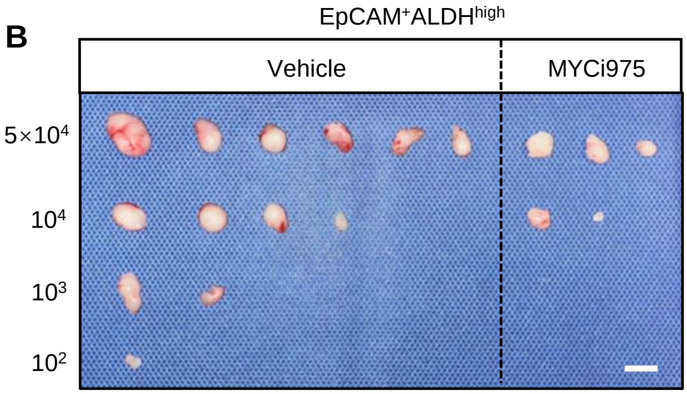

C

Cells injected	Tumor incidence/Number of injection	
	Vehicle	MYCi975
5×10^4	6/6	3/6
10^4	4/6	2/6
10^3	2/6	0
10^2	1/6	0
CSC Frequency 95% CI	1/5701 (1/13694-1/2374)	1/52596 (1/133161-1/20774)
P value	< 0.001	

Figure S5. Related to Figure 3

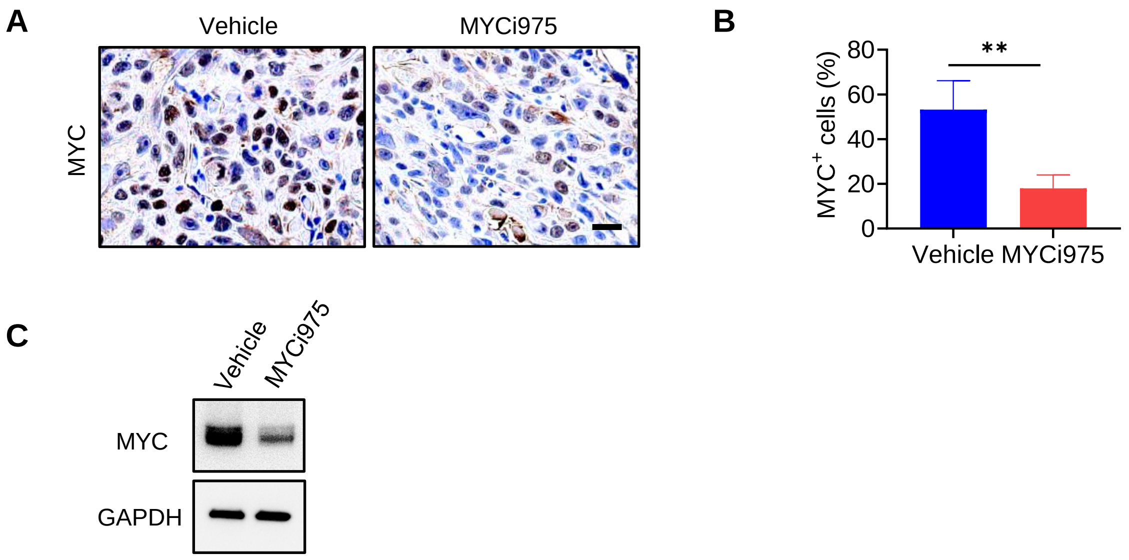

Figure S6. Related to Figure 5

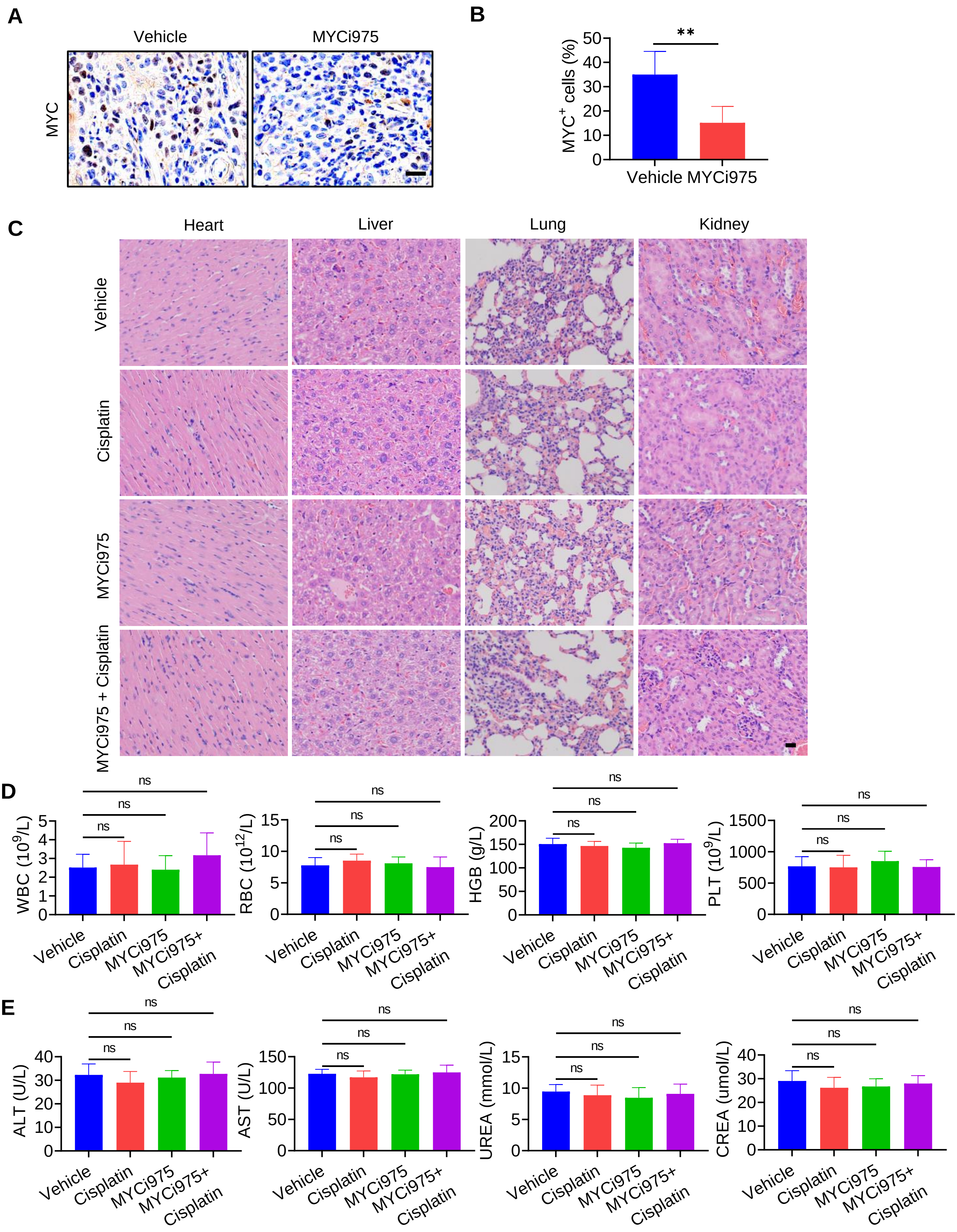

Figure S7. Related to Figure 5

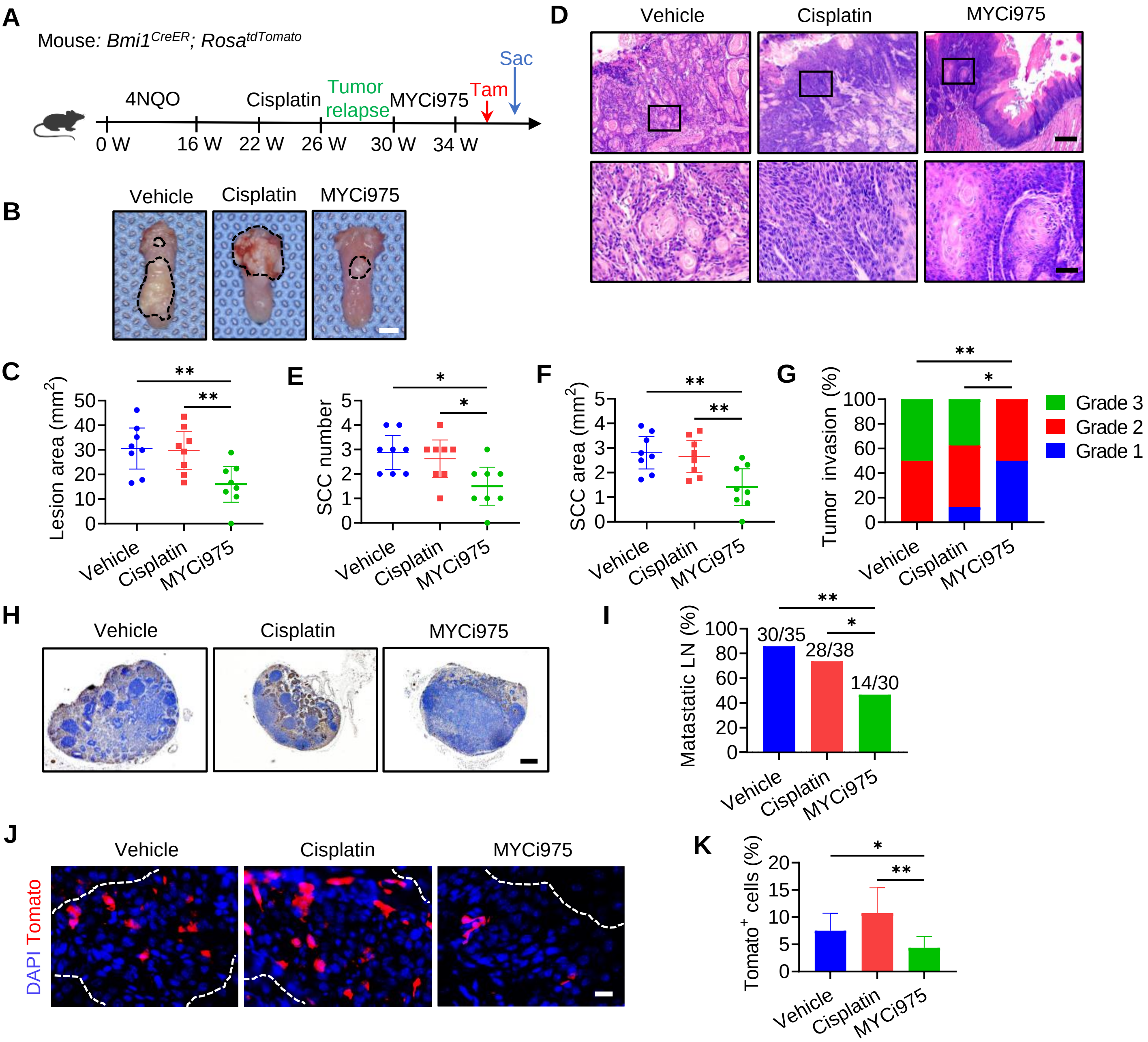

Figure S8. Related to Figure 6

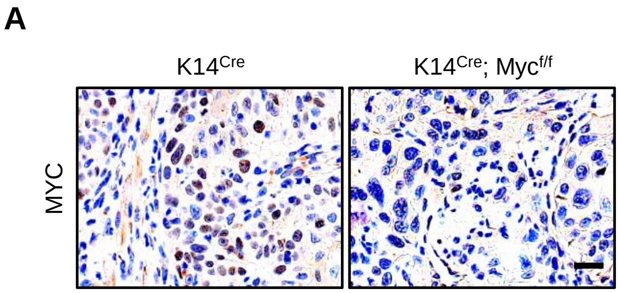

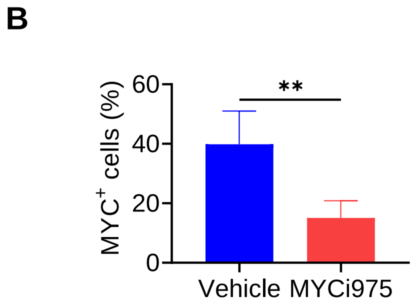

Figure S9. Related to Figure 7

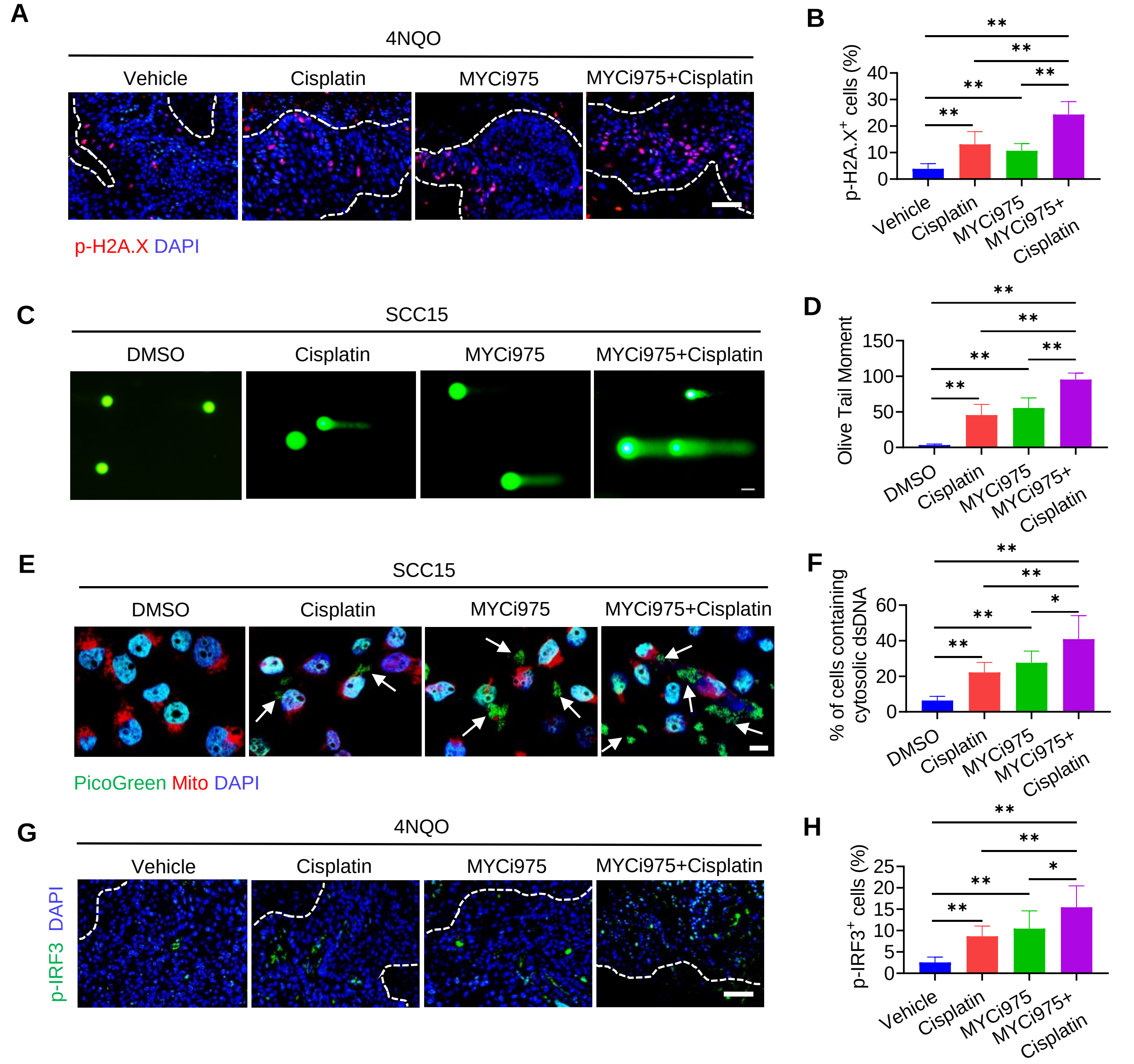

Table S1. Sequences of qRT-PCR primers.

Target genes	Forward (5′-3′)	Reverse (3′-5′)
GAPDH	GGAGCGAGATCCCTCCAAAAT	GGCTGTTGTCATACTTCTCATGG
MYC	GTCAAGAGGCGAACACACAAC	TTGGACGGACAGGATGTATGC
BMI1	CGTGTATTGTTCGTTACCTGGA	TTCAGTAGTGGTCTGGTCTTGT
SOX2	GCCGAGTGGAAACTTTTGTCG	GGCAGCGTGTACTTATCCTTCT
OCT4	TCCCATGCATTCAAACTGAGG	CCTTTGTGTTCCCAATTCCTTCC
NANOG	TCTGCAGAGAAGAGTGTCGC	GGTCTTCACCTGTTTGTAGCTG
KLF4	CCCACATGAAGCGACTTCCC	CAGGTCCAGGAGATCGTTGAA
ALDH1	ACTTACCTGTCCTACTCA	CTTATCTCCTTCTTCTACCT
IFNβ	GCCATCAGTCACTTAAACAGC	GAAACTGAAGATCTCCTAGCCT
CXCL9	CCAGTAGTGAGAAAGGGTCGC	AGGGCTTGGGGCAAATTGTT
CXCL10	GAGCCTACAGCAGAGGAACC	GAGAGGTACTCCTTGAATGCCA
CXCL11	GACGCTGTCTTTGCATAGGC	GGATTTAGGCATCGTTGTCCTTT

Supplemental figure legends:

Figure S1, Related to Figure 1, Different biological characteristics in cisplatin resistant and parental cells

(A) CCK8 assays comparing the proliferation between control and cisplatin-resistant HN6 cells after cisplatin (3 μM) treatment. Means ± SD are shown. **$P < 0.01$ and ns, not significant, using an unpaired Student’s t test. (B) CCK8 assays comparing the proliferation between control and cisplatin-resistant SCC15 cells after cisplatin (3 μM)

treatment. Means ± SD are shown. **P < 0.01 and ns, not significant, using an unpaired Student's t test. (C-D) TUNEL assays (green) comparing apoptosis between control and cisplatin-resistant HN6 cells after cisplatin (3 μM) treatment. Nuclei were stained using DAPI (blue). Means ± SD are shown. **P < 0.01 and ns, not significant using an unpaired Student's t test. (E-F) TUNEL assays (green) comparing of apoptosis between control and cisplatin-resistant SCC15 cells after cisplatin (3 μM) treatment. Nuclei were stained using DAPI (blue). Means ± SD are shown. **P < 0.01 and ns, not significant, using an unpaired Student's t test.

Figure S2, Related to Figure 2, the effect of MYCi975 on the expression of MYC and additional stemness markers in the resistant HNSCC cell lines.

(A) Western blot analysis showing the inhibition of MYC, BMI1, SOX2, and ALDH1 by MYCi975 treatment in cisplatin-resistant HNSCC cell lines. GAPDH was used as the internal control.

Figure S3, Related to Figure 2, MYC inhibition suppresses the cancer stemness of HNSCC cells.

(A) Western blot analysis showing the protein levels of MYC, BMI1, SOX2, and ALDH1 after *MYC* knockdown. GAPDH was used as the internal control. (B-C) Representative FACS plots of $ALDH^{high}$ cells in HN6 and SCC15 cells after *MYC* knockdown. Means ± SD are shown. **P < 0.01 using an unpaired Student's *t* test. A specific inhibitor of ALDH, diethylaminobenzaldehyde (DEAB), was used to control for background fluorescence. (D-G) Images and numbers of tumorspheres formed using $ALDH^{high}$ and $ALDH^{low}$ HN6 and SCC15 cells, with or without *MYC*

knockdown. Scale bar: 100 μm. Means ± SD are shown. $^{**}P < 0.01$ using an unpaired Student's *t* test.

Figure S4, Related to Figure 2, MYCi975 suppresses the cancer stemness in HNSCC cells from human PDX.

(A) Representative FACS plots of $EpCAM^{+}ALDH^{high}$ CSCs from human PDX of HNSCC. (B-C) Extreme limiting dilution analysis (ELDA) of $EpCAM^{+}ALDH^{high}$ tumor cells from HNSCC PDX tissue, with or without MYCi975 treatment *in vivo* (n = 6). The frequency of allograft formation is displayed for each cell dose injected. ELDA software was used to analyze the data. Scale bar: 1 cm.

Figure S5, Related to Figure 3, MYCi975 treatment reduces the protein level of MYC in an orthotopic HNSCC model.

(A) Immunochemistry showing the inhibition of MYC in the orthotopic HNSCC model by MYCi975. Scale bar, 20 μm. (B) Quantification of the percentage of MYC^{+} cells in nude mice orthotopic tumors treated as indicated. The values are means ± SDs from the pool of two independent experiments. n = 10. $^{**}P < 0.01$ using Student's *t* test. (C) Western blot showing the reduction of MYC levels in orthotopic HNSCC tumor tissues after MYCi975 treatment. GAPDH was used as the internal control.

Figure S6, Related to Figure 5, MYCi975 treatment inhibits the protein level of MYC in a 4NQO-induced HNSCC model. MYCi975 and cisplatin tolerability *in vivo*.

(A) Immunochemistry showing the inhibition of MYC in HNSCC by MYCi975. Scale bar, 20 μm. (B) Quantification of the percentage of MYC^{+} cells in HNSCC from

mice treated as indicated. The values are means ± SDs from the pool of two independent experiments. n = 13. $**P < 0.01$ using Student's *t* test. (C) H&E staining of the heart, liver, lung and kidney from the mice treated with control vehicle, cisplatin, MYCi975, MYCi975 plus cisplatin. Scale bar, 20 µm. (D) Comparison of routine blood results among different treatment groups. The white blood count (WBC), red blood count (RBC), Hemoglobin (HGB), and platelet count test (PLT) remained unaffected by cisplatin or MYCi975 treatment. (E) Comparison of blood biochemical indicators between different treatment groups. Alanine aminotransferase (ALT), aspartate aminotransferase (AST), blood UREA, and creatinine (CREA) levels were not significantly affected by cisplatin or MYCi975 treatment. Data represent the mean ± SD from five mice. No significant difference between the control group and treatment group was noted.

Figure S7, Related to Figure 5, MYCi975 treatment overcomes cisplatin secondary resistance in 4NQO-induced HNSCC model.

(A) Experimental design to examine the effect of MYCi975 on cisplatin secondary resistance in a 4NQO-induced HNSCC model. $Bmi1^{CreER}$; $Rosa^{tdTomato}$ mice with 4NQO-induced HNSCC were treated with cisplatin for 4 weeks, and the mice were maintained for additional 4 weeks. Then, mice with recurrent HNSCC were treated with cisplatin, MYCi975 or control vehicle for another 4 weeks. Tamoxifen (Tam) was administered 1 day before sacrificing (Sac) the mice to label $BMI1^+$ CSCs. (B) Representative image of visible tongue lesions in the different treatment groups. Black dashed lines denote the lesion areas. Scale bar, 2 mm. (C) Quantification of the

HNSCC lesion area from mice treated as indicated. The values are means ± SDs from the pool of two independent experiments. n = 8. (D) Representative H&E staining of HNSCC from mice treated as indicated. Scale bar, 200 μm. Enlarged images are shown in the lower panels. Scale bar, 40 μm. (E-F) Quantification of HNSCC tumor number and area from mice treated as indicated. The values are means ± SDs from the pool of two independent experiments. n = 8. $^{*}P < 0.05$ and $^{**}P < 0.01$ using one-way ANOVA. (G) Quantification of HNSCC invasion grades from mice treated as indicated. The data were pooled from two independent experiments. n = 8. $^{*}P < 0.05$ and $^{**}P < 0.01$ using the Cochran-Armitage test. (H) Immunostaining of metastatic cells in cervical lymph nodes using anti-PCK antibodies. Scale bar, 200 μm. (I) Percentage of metastatic lymph nodes from mice treated as indicated. The number of metastatic lymph nodes in each group is indicated in the figure. Data were pooled from two independent experiments. $^{*}P < 0.05$ and $^{**}P < 0.01$ using a χ^2 test. (J) Representative images of Tomato$^+$ BMI1$^+$ CSCs in HNSCC from mice treated as indicated. The white dashed lines denote the tumor-stromal junction. Scale bar, 20 μm. (K) Quantification of the percentage of Tomato$^+$ cells in HNSCC from mice treated as indicated. The values are means ± SDs from the pool of two independent experiments. n = 8. $^{*}P < 0.05$ and $^{**}P < 0.01$ using one-way ANOVA.

Figure S8, Related to Figure 6, *Myc* KO reduces the protein level of MYC in 4NQO-induced HNSCC model.

(A) Immunochemical staining of MYC in HNSCC from both $K14^{Cre}$ and $K14^{Cre}$; $Myc^{f/f}$ mice after Tam treatment. Scale bar, 20 μm. (B) Quantification of the

percentage of MYC^+ cells in HNSCC from mice treated as indicated. The values are means ± SDs from the pool of two independent experiments. n = 11. **$P < 0.01$ using Student's *t* test.

Figure S9, Related to Figure 7, MYCi975 plus cisplatin induces DNA damage and activates the cGAS-STING-IRF3 signaling pathway.

(A-B) Immunostaining and quantification of p-H2A.X (red) in 4NQO-induced HNSCC under different conditions as indicated. Nuclei were stained using DAPI (blue). Means ± SD are shown from three independent experiments. Scale bar, 50 μm. **$P < 0.01$ using one-way ANOVA. (C-D) Representative images and quantification of DNA Comet assays in SCC15 cells treated under different conditions as indicated. More than 100 cells were analyzed per group. Means ± SD are shown. Scale bar, 100 μm. ** $P < 0.01$ using one-way ANOVA. (E-F) Confocal microscopy images showing cytosolic DNA accumulation and quantification in SCC15 cells under different conditions as indicated. Double-stranded DNA (dsDNA) was stained using PicoGreen (green). Mitochondria and nuclei were stained using MitoTracker (Red) and DAPI (blue), respectively. The white arrows indicate cytosolic dsDNA. Scale bar, 10 μm. More than 100 cells were analyzed per group. Means ± SD are shown. ** $P < 0.01$ using one-way ANOVA. (G-H) Immunostaining and quantification of p-IRF3 (green) in 4NQO-induced HNSCC under different conditions as indicated. Nuclei are stained using DAPI (blue). Means ± SD are shown from three independent experiments. Scale bar, 50 μm. *$P < 0.05$ and ** $P < 0.01$ using one-way ANOVA.
